# Direct anterior approach versus posterior approach in total hip arthroplasty: A systematic review and meta-analysis

**DOI:** 10.1016/j.jor.2025.06.007

**Published:** 2025-06-14

**Authors:** Maher Ghandour, Ouriel Salomon, Massinissa Hammouchi, Aboubacar Lawan Abdou, Kevin Okoma, Meriem Souissi, Dounia Brahimi, Lisa Gosnave, Ümit Mert, Mauz Asghar, Julien Mayer, Emmanuel Caremier, Aboubekr Berrichi, M'barek Irrazi

**Affiliations:** a Orthopedic and Traumatology Department, CHR Metz-Thionville, Metz, France; b Department of Trauma and Orthopaedic Surgery, Helios University Hospital Wuppertal, University of Witten/Herdecke, Wuppertal, Germany; c University of Saskatchewan College of Medicine, Saskatchewan, Canada

**Keywords:** Total hip arthroplasty, Direct anterior approach, Posterior approach, Minimally invasive surgery, Harris hip score

## Abstract

**Background:**

The optimal surgical approach for total hip arthroplasty (THA) is still debated. Recently, a growing surgical trend toward the utilization of minimally invasive techniques, such as the direct anterior approach (DAA), has emerged in the THA area. However, there are ongoing concerns regarding its technical complexity and perioperative outcomes relative to the posterior approach (PA). Therefore, we aim to compare DAA and PA regarding perioperative, functional, and safety outcomes.

**Methods:**

A comprehensive systematic search of PubMed, Web of Science, Scopus, and Cochrane Library was executed. We included randomized controlled trials (RCTs) and observational studies comparing DAA and PA in patients undergoing THA. The primary endpoints were all-cause surgery revision, dislocation, and fracture. Secondary endpoints encompassed the duration of hospital stay, incision length, functional recovery measured using the Harris Hip Score (HHS), and complications. Mean difference (M.D.) or Risk ratio (R.R.) with a 95 % confidence interval (C.I.) were employed to analyse the continuous or dichotomous outcomes.

**Results:**

27 studies constituted our meta-analysis, including 44,477 patients**,** with 7138 patients in the DAA cohort and 37,299 patients in the PA cohort. Our pooled analysis demonstrated comparable estimates of all-cause surgery revision (R.R. = 0.90, 95 % C.I. [0.71, 1.15], p = 0.40), dislocation (R.R. = 0.78, 95 % C.I. [0.53, 1.16], p = 0.22), intraoperative fracture (R.R. = 0.85, 95 % C.I. [0.51, 1.42], p = 0.54), and periprosthetic fracture (R.R. = 2.14, 95 % C.I. [0.85, 5.38], p = 0.11). Notably, DAA showed a significantly shorter hospital stay (M.D. = −0.31 days, 95 % C.I. [-0.55, −0.07], p = 0.01) and shorter incision length (M.D. = −33.75 mm, 95 % C.I. [-42.97, −24.54], p = 0.001) compared to the PA. No significant variations were noticed between the two approaches regarding HHS.

**Conclusion:**

Our meta-analysis highlighted that DAA is effective as PA in mitigating the risk of major complications following THA, such as all-cause surgery revision, dislocation, and fracture. In contrast, DAA showed better perioperative results, including shorter hospital stays and incision lengths, without compromising the safety outcomes compared to PA.

## 1 Introduction

Total hip arthroplasty (THA) documented 50 % incidence increase between 1990 and 2002 and projections estimating 572,000 annual procedures by 2030.^1^^,^^2^ The primary goal of THA is to replace the damaged femoral head and acetabulum with artificial implants to restore joint function, relieve pain, and enhance the quality of life.^3^ Nevertheless, around 7–15 % of patients showed dissatisfaction with THA owing to persistent postoperative pain and late functional recovery.^4^^,^^5^

Failure of fixation and the substantial damage to soft tissue are considered the most significant causes for this postoperative pain.^6^ One of the critical contributors to soft tissue damage is the surgical approach utilized.^7^^,^^8^ Notably, the ideal surgical approach for THA is still inconclusive, and the selection of surgical modality largely depends on the surgeon's experience and individual patient characteristics.^9^ The posterior approach (PA) was traditionally considered the preferred modality for THA worldwide.^10^ However, PA is an invasive approach and is associated with significant tissue damage that could compromise the surgical results and delay the patient's recovery.^11^

Nowadays, there is an increasing trend toward implementing minimally invasive techniques as they are associated with less tissue damage and faster postoperative recovery. Therefore, the direct anterior approach (DAA) for THA has become popular recently among orthopaedic surgeons.^12^ DAA relies on an intermuscular plane to insert components, which could result in reduced soft tissue damage and improve the operative outcomes.^8^ Several studies have shown a greater benefit for DAA over PA regarding blood loss, hospital stay, and functional recovery.^12^, ^13^, ^14^, ^15^ However, other reports have raised concerns about its higher risk of complications, such as fracture and component malpositioning, compared to other modalities.^16^^,^^17^ Therefore, we executed this study to address this knowledge gap and provide a comprehensive comparison between DAA and PA regarding perioperative, functional, and complication outcomes.

## 2 Materials and methods

This study was carried out in full accordance with the Cochrane Handbook recommendations and reported in line with PRISMA guidelines.^18^^,^^19^

### 2.1 Criteria for inclusion

Our study included Randomized controlled trials (RCTs) and observational studies that met our predefined eligibility criteria. The target population was patients undergoing THA. DAA represented the intervention group, while the PA was the comparator group. The main outcomes were all-cause surgery revision, revisions due to particular complications, dislocation, and fracture. Secondary perioperative outcomes included operative time, duration of hospital stay, incision length, blood loss volume, the need for blood transfusion, and discharge status, either to home or rehabilitation.

We also studied the functional outcomes assessed by several scores, including Oxford Hip Score (OHS), Western Ontario and McMaster Universities Osteoarthritis (WOMAC), Harris Hip Score (HHS), Health Survey Short Forms (SF-12) Physical score, and Health Survey Short Forms (SF-12) Mental score. The Visual Analogue Scale (VAS) was implemented to assess pain. Complications evaluated were surgical site hematoma and infection.

Our exclusion criteria were studies comparing DAA and PA in patients undergoing hemiarthroplasty, animal studies, foreign language studies, and conference abstracts.

### 2.2 Search process and screening

The scope of our electronic search encompassed PubMed, Scopus, Web of Science, and Cochrane Library from inception until April 2025. Our search strategy included all available DAA, PA, and THA keywords. In-depth information regarding the search strategy, filters applied, and the number for each database is presented in Supplementary Table 1.

The retrieved articles from the electronic search were imported into EndNote (Clarivate Analytics, PA, USA) to identify and remove duplicates. Two independent authors evaluated the retrieved studies in a two-step screening process. First, the titles and abstracts of the eligible articles were extensively evaluated using Rayyan Web.^20^ Then, the full text of eligible articles was assessed employing Google Sheets. An additional screening of the references of the eligible studies was conducted to ensure the selection process. Any disagreements that appeared during the screening process were addressed through discussion.

### 2.3 Data gathering

Utilizing a predesigned data collection sheet, two blinded authors extracted the subsequent data, including the study design, country, sample size, time frame, indications of THA, inclusion criteria, main findings, age, body mass index (BMI), Sex, ASA class, and HHS. A third independent author resolved disagreements among the review authors during the data extraction process.

### 2.4 Risk of bias

Two blinded reviewers utilized the Cochrane risk of bias two (ROB 2) tool to assess the risk of bias of RCTs.^21^ ROB2 tool assesses the methodology of each RCT regarding randomization process, deviation from the intended intervention, missing outcome data, measurement of the outcome, selection of the reported results, and other sources of bias. Each study was appraised and assigned to one of these categories: low, some concerns, or high risk of bias. The Newcastle Ottawa scale (NOS) was also implemented to evaluate the risk of bias of the observational studies.^22^ The NOS assesses three main aspects: population and exposure selection, comparability between cohorts, and outcome assessment. NOS categorized each study as having good, fair, or low quality according to the cumulative score.

### 2.5 Data synthesis

Mean difference (MD) with 95 % confidence interval (CI) was utilized to pool the continuous outcomes, while the risk ratio (RR) with 95 % CI was used to synthesize the dichotomous outcomes. The DerSominian Laird random effect meta-analysis model was implemented with a p-value <0.05, suggesting statistical significance. The significant heterogeneity was identified when the chi-square p-value was less than 0.1 or I^2^ > 50 %. A leave-one-out test was performed for the primary outcomes to ensure that the pooled estimate was not dependent on any individual study. A funnel plot was used to represent the relationship between the pooled estimate and standard error to investigate the publication bias. All the statistical analysis was carried out using STATA 18 BE for MAC.

## 3 Results

### 3.1 Literature search

**Fig. 1:**
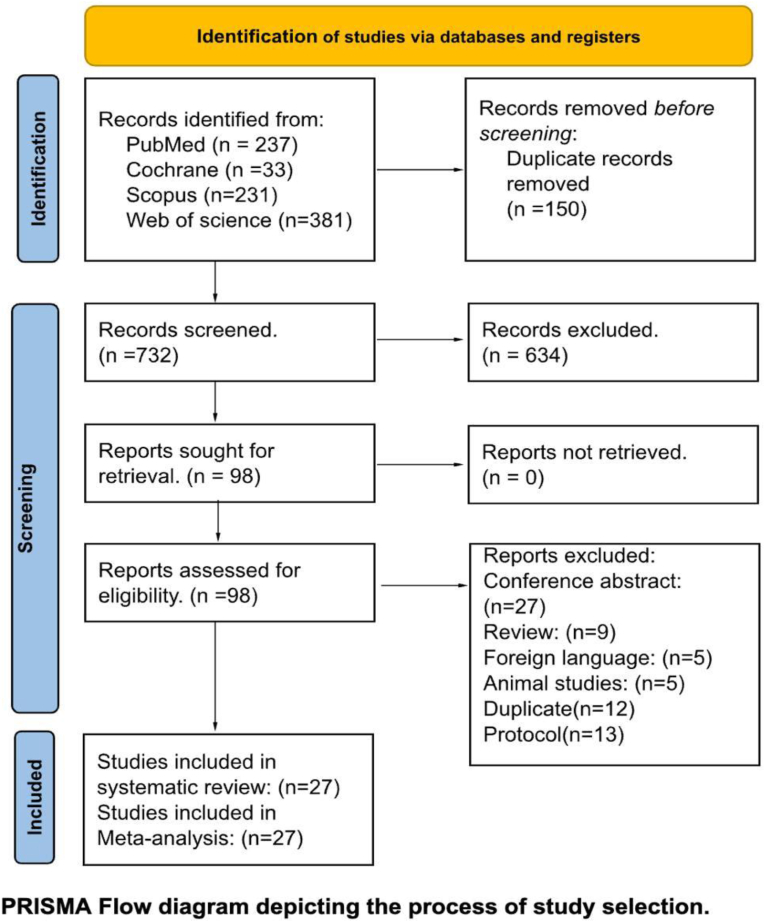
PRISMA flow diagram of the study selection process.

The initial search of the four databases yielded 882 articles, 150 of which were eliminated as duplicates, resulting in 732 articles available for the title and abstract screening phase. 634 articles were eliminated in this phase, with 98 articles proceeding to the full-text screening stage. Consequently, 71 articles were eliminated in this stage, resulting in 27 studies finally included in this study, as illustrated in Fig. 1.

### 3.2 Studies characteristics

This study encompassed 27 studies, incorporating 44,477 patients who underwent THA, with 7138 managed by the DAA, and 37,299 received the PA.^11^^,^^14^^,^^15^^,^^23^, ^24^, ^25^, ^26^, ^27^, ^28^, ^29^, ^30^, ^31^, ^32^, ^33^, ^34^, ^35^, ^36^, ^37^, ^38^, ^39^, ^40^, ^41^, ^42^, ^43^, ^44^, ^45^^,^^45^^,^^46^ Most included studies were cohort studies (n = 17), while the remaining 10 were RCTs. Geographically, twenty studies were conducted in the USA, six in several European countries, and one in Asia, particularly Japan. Osteoarthritis was the most common indication for THA across the included studies. The mean follow-up period was 19.2 months, varying from 1.5 to 60 months. Furthermore, the average age of the study population was 61.4 [3.6] years. Detailed information about the properties of studies is presented in Table 1.

**Table 1:** Summary and baseline characteristics of the included studies.

Study ID	Study arms	Study Design	Country	Sample Size			Folow-up	Time Frame	Indications of THA	Main Finding	Age (Years)	BMI, kg/m2	Sex		ASA class	HHS
													Male	Female		
				Total	DDA	PA					Mean (SD)	Mean (SD)	N (%)	N (%)	Mean (SD)	Mean (SD)
Barrett 2019	**DAA**	RCT	United States	87	43	44	5 years	January 2010 to April 2011	NIDJD as Osteoarthritis, DDH and AVN	No significant differences in survivorship, function (Harris hip score, UCLA activity score, HOOS Jr), complications, or radiographic outcomes between DAA and PA at 5-year follow-up.	NA	NA	NA	NA	NA	56.42 (10.42)
	**PA**										NA	NA	NA	NA	NA	53.8 (10.19)
Bon 2019	**DAA**	RCT	France	108	54	54	3 months	February 2017 to April 2018	Hip osteoarthritis	No significant differences in early functional recovery, gait parameters, or functional scores. Operative time was significantly longer in the DAA group and Higher rate of lateral cutaneous nerve neuropraxia in the DAA group.	67.26 (10)	26.46 (3.58)	21 (38.9)	29 (61.1)	NA	54.04 (14.94)
	**PA**										68.98 (7.93)	26.69 (3.12)	23 (42.5)	27 (57.5)	NA	52.31 (13.06)
Cheng 2016	**DAA**	RCT	Australia	73	35	38	3 months	March 2014 to March 2015	Hip osteoarthritis	DAA had shorter acute hospital stay, smaller wounds, and less postoperative opiate use but longer operative times, higher blood loss, and weaker hip flexion initially.	60.67 (11.59)	27.83 (3.25)	15 (43)	20 (57)	NA	NA
	**PA**										62.17 (10.79)	28.07 (4.85)	18 (47)	20 (53)	NA	NA
Christensen 2015	**DAA**	RCT	United States	53	29	24	6 weeks	NA	NA	DAA resulted in shorter hospital stays, earlier discontinuation of assistive devices, and greater pain relief. No significant differences in other functional outcomes between DAA and PA.	64.3 (9.1)	31.1 (5.1)	13 (44.8)	15 (45.2)	NA	NA
	**PA**										65.2 (9.1)	30.4 (3.6)	11 (0.45)	12 (55)	NA	NA
Moerenhout 2020	**DAA**	RCT	Canada	55	28	27	55 months	February 2011 to July 2013	osteoarthrosis or osteonecrosis	DAA had a significantly longer surgical time. No significant differences in hospital length of stay, functional outcome, pain, implant position, or complications between DAA and PA. DAA showed a trend toward better functional recovery in the first 3 months.	70.4 (9.1)	27.6 (4.4)	11 (39.2)	17 (60.8)	1.8 (0.7)	52.1 (19.7)
	**PA**										68.9 (8.8)	26.5 (4.3)	18 (66.7)	9 (33.3)	2 (0.8)	48.2 (10.1)
Nambiar 2021	**DAA**	RCT	Australia	73	35	38	60 months	NA	Osteoarthritis	No differences in functional (OHS, WOMAC), quality-of-life (EQ-5D), or radiographic outcomes. Implant survivorship was 97 % for both approaches. DAA had a higher incidence of lateral femoral cutaneous nerve (LFCN)	64 (11)	27 (3)	23 (66)	12 (34)	NA	NA
	**PA**										66 (10)	28 (4)	22 (58)	16 (42)	NA	NA
Roberts 2024	**DAA**	RCT	United States	101	52	49	90 months	March 2013 to May 2016	NA	No clinically meaningful differences in patient-reported outcomes, complications, reoperations, or revisions between DAA and MPA at 7.5 years. Early functional benefits of DAA were not sustained beyond 8 weeks	65	29	27 (51)	25 (49)	NA	NA
	**PA**										64	39	25 (51)	24 (51)	NA	NA
Rodriguez 2014	**DAA**	RCT	United States	132	67	65	12 months	January to December 2010	Osteoarthrosis	DAA showed modest functional advantages in TUG and M-FIM™ scores up to 2 weeks postoperatively compared to the PA. No differences were observed between the groups beyond 6 weeks in terms of functional recovery, general health outcomes, operative time, complications, or component alignment.	60 (10)	27 (4)	28 (46.6)	32 (53.4)	NA	49.4 (7.5)
	**PA**										59 (6)	28 (4)	26 (43)	34 (57)	NA	46.6 (11.5)
Taunton 2014	**DAA**	RCT	United States	54	27	27	12 months	January 2012 to August 2012	Primary degenerative arthritis of the hip	Patients undergoing direct anterior approach (DAA) THA voluntarily discontinued all walking aids 6 days earlier on average compared to those with mini-posterior approach (MPA) THA. No significant differences in most clinical or radiographic outcomes between the two approaches.	62.05	27.7	12 (44.5)	15 (55.5)	NA	54.67 (13.3)
	**PA**										66.4	29.2	13 (48.1)	14 (51.9)	NA	55 (18.78)
Taunton 2018	**DAA**	RCT	United States	101	52	49	12 months	March 2013 to May 2016	Osteoarthritis	The DAA group showed slightly faster early functional recovery (e.g., discontinued gait aids earlier: 17 vs. 24 days for MPA). No differences in patient-reported outcomes (HOOS, SF-12, Harris hip scores), radiographic outcomes, or complications at 1 year. Both approaches provided excellent recovery with low complication rates.	65 (10)	29 (22)	27 (51)	25 (49)	NA	57 (13)
	**PA**										64 (11)	30 (4)	25 (51)	24 (49)	NA	56 (12)
Balasubramaniam 2016	**DAA**	Retrospective cohort study	Australia	92	50	42	12 months	January 2006 to December 2011	Osteoarthritis (OA) or avascular necrosis (AVN)	DAA showed improved HHS at 12 months, shorter hospital stays, shorter operating times, and lower return-to-theatre rates compared to the posterior approach (PA).	62.5 (9.01)	31.3 (5.2)	30 (60)	20 (40)	2.2 (0.7)	38.2 (11.5)
	**PA**										57 (12.84	29.9 (6.7)	14 (33.4)	28 (66.6)	2.2 (0.7)	35.2 (12.3)
Bergin 2011	**DAA**	Prospective cohort study	United States	57	29	28	1 month	NA	Osteoarthritis	The anterior approach caused significantly less muscle damage (measured by serum CK levels) compared to the posterior approach. Inflammatory marker levels (CRP, IL-6, TNF-α, IL-1β) were similar between groups, suggesting comparable physiologic burden. No functional outcomes were assessed; relevance of CK differences remains to be determined.	68.8 (9.1)	26.3 (5)	10 (34)	19 (66)	2.(0.6)	42.4 (6)
	**PA**										65.1 (11.3)	27.8 (5)	14 (50)	14 (50)	1.9 (0.5)	43.0 (11)
Cochrane 2024	**DAA**	Retrospective cohort study	United States	2101	1009	1092	3 months	January 2017 to December 2022	NA	The DAA was independently associated with higher encounter and 90-day costs compared to the PA, despite the DAA cohort being younger and healthier. No significant difference in clinical outcomes, but DAA had higher ED visits and readmissions.	55.5 (11.45)	30.22 (4.7)	428 (42.4)	581 (57.6)	2.4 (0.46)	NA
	**PA**										58.75 (11.22)	32.7 (6.24)	546 (50)	546 (50)	2.46 (0.46)	NA
Hamilton 2015	**DAA**	Retrospective cohort study	United States	200	100	100	NA	NA	Osteoarthritis, rheumatoid arthritis and hip dysplasia	The DAA group had significantly less variance in cup anteversion, and abduction angles compared to the PA group. The use of intraoperative fluoroscopy in DAA allowed for more accurate cup placement and eliminated severely vertical cups (>55° abduction) seen in the PA group.	61.7 (11.6)	30.8(5.42)	36 (36)	64 (64)	NA	NA
	**PA**										60.8 (10.2)	32.2 (4.92)	39 (39)	61 (61)	NA	NA
Maldonado 2019	**DAA**	Prospective cohort study	United States	48	24	24	3 months	March 2014 to October 2017	Osteoarthritis	DAA showed superior early outcomes at 3 months compared to PA, with higher scores in HHS, VR-12 Mental/Physical, SF-12 Physical, and lower VAS pain scores. No difference in patient satisfaction or FJS-12.	58.9 (11.1)	30.9 (6.2)	6 (25)	18 (75)	NA	NA
	**PA**										60.1 (9.3)	31.2(5.6)	6 (25)	18 (75)	NA	NA
Maldonado 2021	**DAA**	Prospective cohort study	United States	410	205	205	24 months	July 2008 to July 2016	Osteoarthritis	Both DAA and PA groups had favorable outcomes at 2-year follow-up. DAA showed superior quality-of-life outcomes (VR-12 Mental/Physical, SF-12 Mental/Physical) but comparable HHS, FJS-12, VAS, and satisfaction scores. No significant difference in complications or revisions between groups.	59.3 (8.8)	29.4 (4.6)	86 (42)	119 (58)	NA	NA
	**PA**										58.8 (10.5)	29.6 (4.7)	86 (42)	119 (58)	NA	NA
Malek 2016	**DAA**	Retrospective cohort study	United Kingdom	488	265	183	18 months	December 2010 to February 2014	NA	No significant difference in clinical outcomes (length of stay, pain scores, OHS, complications) between DAA and PA when ERAS is used. Higher rate of periprosthetic femoral fractures in the DAA group.	69.9 (7.08)	30.7 (5.8)	117 (45.7)	148 (54.3)	2.3 (0.53)	NA
	**PA**										66.5 (13.67)	31.65 (5.6)	86 (47)	97 (53)	2.3 (0.6)	NA
Marat 2016	**DAA**	Retrospective cohort study	United States	4294	2147	2147	3 months	February 2012 to September 2014	NA	No significant difference in dislocation rates between DAA and PA. Neither approach demonstrated a compelling advantage in short-term outcomes or complications.	64.36 (10.93)	28.97 (5.51)	978 (46)	1169 (54)	NA	NA
	**PA**										64.84 (12.08)	29.30 (5.01)	1030 (48)	1117 (52)	NA	NA
Nakata 2009	**DAA**	Retrospective cohort study	Japan	195	99	96	12 months	May 2003 to December 2006	Osteoarthritis, osteonecrosis, and rapidly destructive coxarthritis	Faster functional recovery (earlier single-leg stance, fewer Trendelenburg signs, improved walking ability at 3 weeks). Better cup positioning accuracy (99 % within safe zone vs. 91 % in MPA)	62.9 (11.94)	22.9 (3.98)	16 (16)	83 (84)	NA	NA
	**PA**										65.6 (11.75)	23.3 (3.91)	13 (13.5)	83 (86.5)	NA	NA
Poehling-Monaghan 2015	**DAA**	Retrospective cohort study	United States	222	126	96	2 months	April 2011 to March 2012	NA	No systematic advantage of DAA over PA in early recovery, complications, or component positioning. DAA had longer operative times, higher early pain scores, and more gait aid use at 2 weeks, but fewer minor wound problems. PA patients had a higher proportion free of gait aids at 2 weeks and returned to work/driving sooner at 8 weeks.	64.8 (12.4)	30 (5.5)	59 (46)	67 (53)	NA	55.4 (10.5)
	**PA**										63.9 (12.5)	30.5 (6.0)	52 (54)	44 (45)	NA	56.4 (14.3)
Torres-Ramirez 2024	**DAA**	Retrospective cohort study	United States	252	126	126	37 months	February 2016 to April 2019	Osteoarthritis	DAA advantages: Shorter surgical time, anesthesia time, and hospital stay, and higher discharge-to-home rate. Safety: No differences in complications (in-hospital, 90-day, or reoperations) or patient-reported outcomes (HOOS-JR scores) between approaches.	52.75 (11.7)	30.75 (6)	58 (46)	68 (54)	NA	NA
	**PA**										52.75 (9.9)	28.7 (4.84)	58 (46)	68 (54)	NA	NA
Rathod 2014	**DAA**	Prospective cohort study	United States	22	11	11	12 months	PA group: June 2008–June 2009 DAA group: April 2010–June 2011	Osteoarthritis	Both DAA and PA THA showed similar improvement in most gait parameters The DAA group had significantly better internal/external rotation ROM postoperatively, possibly due to external rotator release/repair in PA. No differences in clinical outcomes (Harris Hip Scores) between groups.	58 (6.7)	25.9 (2.33)	6 (54.4)	5 (45.6)	NA	54.84 (10.5)
	**PA**										61.8 (9.1)	25.34 (3.08)	6 (54.4)	5 (45.6)	NA	53.32 (9)
Richards 2024	**DAA**	Prospective cohort study	United States	65	34	31	1 month	April 2018 to March 2020	NA	BRT returned to baseline by 2 weeks post-THA in both groups. The PA group showed quicker return to preoperative BRT at 1 week compared to DAA. By 4 weeks, 93 % of PA patients returned to baseline BRT vs. 60 % in DAA. No differences in BPD (strength) or HOOS JR scores between approaches.	55.8	NA	21 (61.7)	13 (38.3)	NA	NA
	**PA**										57.8	NA	21 (67.7)	10 (32.3)	NA	NA
Sarhan 2024	**DAA**	Prospective cohort study	United States	1364	524	840	12 months	2018 to 2022	Osteoarthritis	DAA showed earlier improvements in HOOS JR and EQ-5D-5L scores up to 6 months post-operatively, but differences were not clinically significant and disappeared by 1 year.	61.8 (9.9)	28.9 (5.7)	225 (42.94)	299 (57.06)	NA	NA
	**PA**										61.3 (10.6)	29.6 (5.8)	408 (48.57)	432 (51.43)	NA	NA
Sheth 2015	**DAA**	Retrospective cohort study	United States	33598	1851	31747	36 months	April 2001 to December 2011	NA	Anterior had a lower risk of dislocation compared to the posterior approach, without increasing the risk of early revision. No differences in aseptic or septic revision rates across approaches.	65 (11)	28 (5)	736 (40)	1112 (60)	NA	NA
	**PA**										66 (12)	29 (6)	13,478 (43)	18,264 (58)	NA	NA
Tripuraneni 2016	**DAA**	Retrospective cohort study	United States	132	66	66	14 months	April 2012 to August 2015	Osteoarthritis (majority), inflammatory arthritis, rheumatoid arthritis, Crowe I/II hip dysplasia, osteonecrosis, or post-arthroscopy degeneration.	No significant difference in acetabular component position (abduction/anteversion) or dislocation frequency between DAA and PA. DAA dislocation rate: 3.0 % vs. PA: 1.5 %, statistically insignificant. Fluoroscopy in DAA did not improve cup positioning over free-hand PA	60.2	27.6	26 (39.3)	40 (60.7)	NA	NA
	**PA**										60.2	27.8	26 (39.3)	40 (60.7)	NA	NA
Zawadsky 2014	**DAA**	Retrospective cohort study	United States	100	50	50	6 weeks	NA	Osteoarthritis or avascular necrosis.	DAA had shorter hospital stay, Higher discharge to home, less narcotic use at 6 weeks, Faster mobility recovery.	60.8 (11.8)	28.6 (6.2)	22 (44)	28 (56)	2.3 (0.51)	NA
	**PA**										56 (11.4)	27.9 (6.2)	14 (28)	36 (72)	2.2 (0.51)	NA

Abbreviations: DAA: Direct anterior approach, PA: Posterior approach, THA: Total hip arthroplasty, BMI: Body mass index, ASA: American Society of Anaesthesiologists, HHS: Harris hip score, SD: Standard deviation, NA: Not applicable.

### 3.3 Risk of bias results

**Fig. 2:**
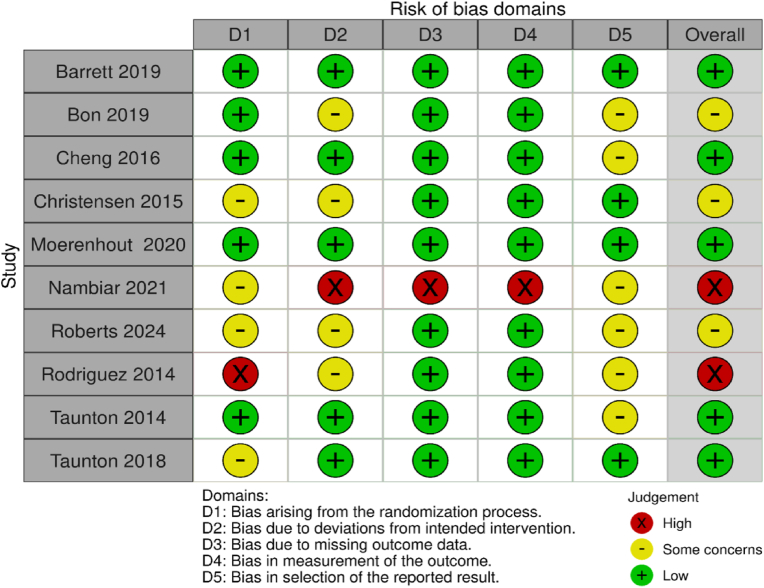
Risk of bias of randomized controlled trials.

The 10 RCTs evaluated by ROB2 showed a variety of risk of bias, with five studies judged as having a low risk of bias, three showing some concerns, and two showing a high risk of bias, as detailed in Fig. 2. The NOS judged 16 studies as having good quality, while the remaining study revealed a fair quality, as outlined in Table 2.

**Table 2:** Risk of bias of observational studies using the Newcastle Ottawa scale.

Study ID	Selection				Comparability	Outcome			Quality Score
	Representativeness of the exposed cohort	Selection of the non-exposed cohort	Ascertainment of exposure	Demonstration that outcome of interest was not present at start of study	Comparability of cohorts on the basis of the design or analysis	Assessment of outcome	Was follow-up long enough for outcomes to occur	Adequacy of follow up of cohorts	
Balasubramaniam 2016	**∗**	**∗**	**∗**	**∗**	**∗**	**∗**	**∗**	**∗**	**Good**
Bergin 2011	**∗**	**∗**	**∗**	**∗**	**∗∗**	**∗**		**∗**	**Good**
Cochrane 2024	**∗**	**∗**	**∗**	**∗**	**∗**	**∗**	**∗**	**∗**	**Good**
Hamilton 2015	**∗**	**∗**	**∗**	**∗**	**∗**	**∗**			**Fair**
Maldonado 2019	**∗**	**∗**	**∗**	**∗**	**∗∗**	**∗**	**∗**	**∗**	**Good**
Maldonado 2021	**∗**	**∗**	**∗**	**∗**	**∗**	**∗**	**∗**	**∗**	**Good**
Malek 2016	**∗**	**∗**	**∗**	**∗**	**∗**	**∗**	**∗**	**∗**	**Good**
Maratt 2016	**∗**	**∗**	**∗**	**∗**	**∗∗**	**∗**	**∗**	**∗**	**Good**
Nakata 2009	**∗**	**∗**	**∗**	**∗**	**∗∗**	**∗**	**∗**	**∗**	**Good**
Poehling-Monaghan 2015	**∗**	**∗**	**∗**	**∗**	**∗∗**	**∗**		**∗**	**Good**
Torres-Ramirez 2024		**∗**	**∗**	**∗**	**∗∗**	**∗**	**∗**		**Good**
Rathod 2014	**∗**	**∗**	**∗**	**∗**	**∗**	**∗**	**∗**		**Good**
Richards 2024	**∗**	**∗**	**∗**	**∗**	**∗∗**	**∗**	**∗**	**∗**	**Good**
Sarhan 2024	**∗**	**∗**	**∗**	**∗**	**∗**	**∗**	**∗**		**Good**
Sheth 2015	**∗**	**∗**	**∗**	**∗**	**∗∗**	**∗**	**∗**	**∗**	**Good**
Tripuraneni 2016		**∗**	**∗**	**∗**	**∗∗**	**∗**		**∗**	**Good**
Zawadsky 2014		**∗**	**∗**	**∗**	**∗∗**	**∗**		**∗**	**Good**

### 3.4 Study outcomes

#### 3.4.1 Primary outcomes

**Fig. 3:**
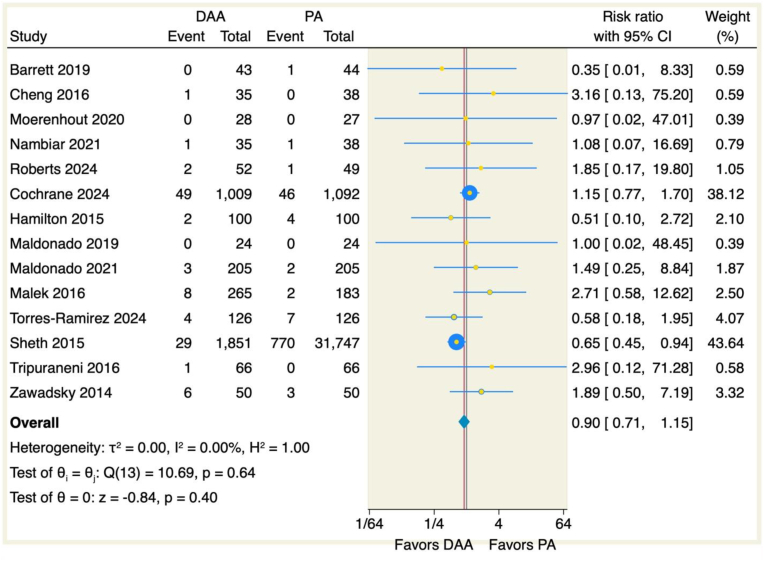
Forest plot of all-cause surgery revision.

14 studies reported the all-cause surgery revision**,** encompassing 3889 patients in the DAA group and 33,789 patients in the PA group. The incidence of all-cause surgery revision was 2.7 % (106/3889) in the DAA group and 2.5 % (837/33,789) in the PA group. The pooled analysis demonstrated similar rates of all-cause surgery revision between the two approaches (R.R. = 0.90, 95 % C.I. [0.71, 1.15], p = 0.40), with homogenous pooled results (I^2^ = 0 %, p = 0.64), as outlined in Fig. 3. The leave-one-out test ensured this robust analysis, as no single study was noticed to have a determinant impact on the pooled estimate, as displayed in Supplementary Fig. 1. Furthermore, minor asymmetry was detected upon inspection of the funnel plot, indicating a small likelihood of publication bias regarding all-cause surgery revision, as shown in Supplementary Fig. 3.

**Fig. 4:**
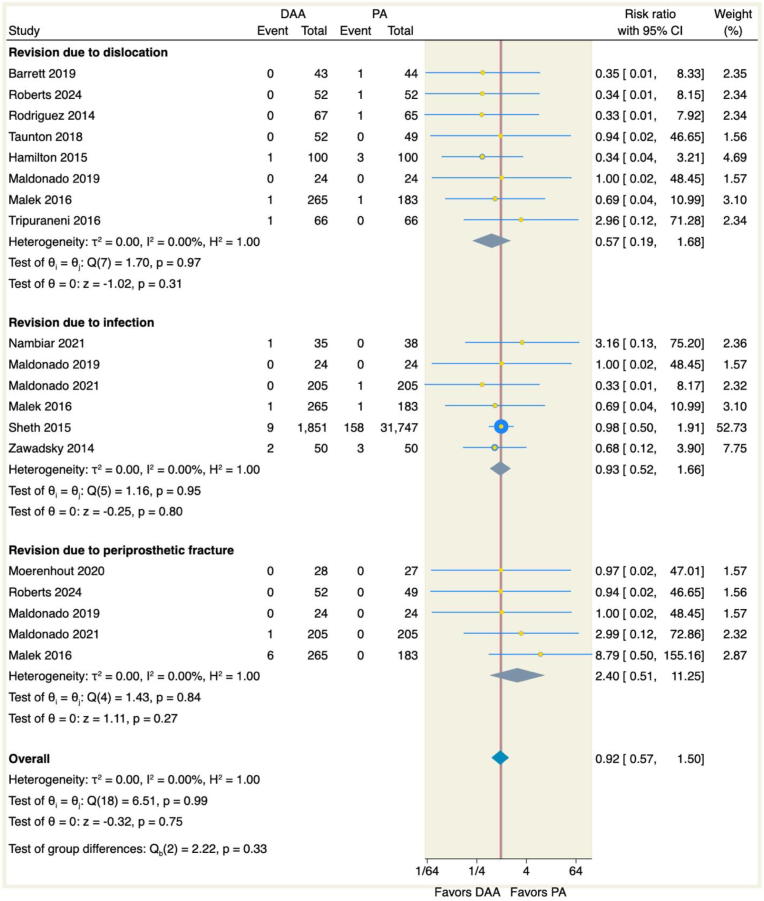
Forest plot of surgery revision due to specific complications.

In the same context, no significant differences between the two approaches were detected in terms of surgery revision due to dislocation (R.R. = 0.57, 95 % C.I. [0.19, 1.68], p = 0.31), surgery revision due to infection (R.R. = 0.93, 95 % C.I. [0.52, 1.66], p = 0.80), or surgery revision due to periprosthetic fracture (R.R. = 2.4, 95 % C.I. [0.51, 11.25], p = 0.27), as displayed in Fig. 4.

**Fig. 5:**
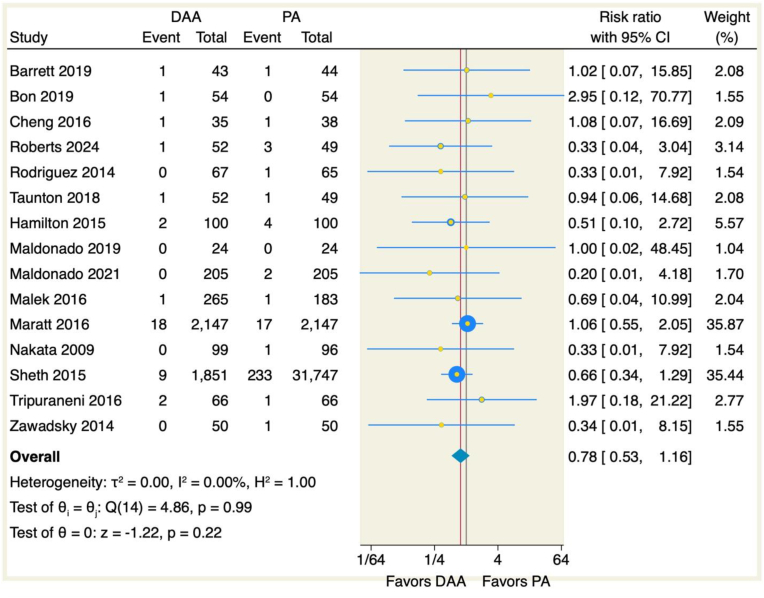
Forest plot of dislocation.

The dislocation rates were reported in 15 studies, with an incidence rate of 7 % (37/5110) in the DAA cohort and 7.6 % (267/34,917) in the PA cohort. The pooled analysis revealed comparable dislocation rates between the two approaches (R.R. = 0.78, 95 % C.I. [0.53, 1.16], p = 0.22), with homogenous combined results (I^2^ = 0 %, p = 0.99), as outlined in Fig. 5. No significant effect was found for any individual study on the pooled estimate, upon conducting the leave-one-out test, as illustrated in Supplementary Fig. 2**.** The funnel plot exhibited a minor asymmetry around the pooled estimate, suggesting a minimal degree of publication bias regarding dislocation, as shown in Supplementary Fig. 4.

**Fig. 6:**
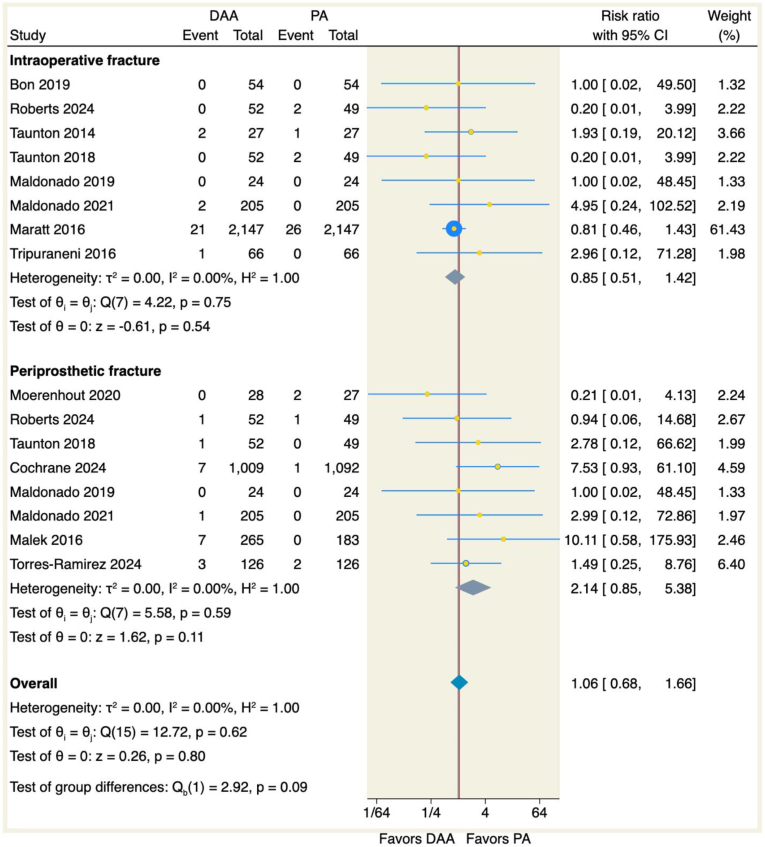
Forest plot of fracture.

Additionally, the pooled analysis revealed similar rates of intraoperative fracture (R.R. = 0.85, 95 % C.I. [0.51, 1.42], p = 0.54) and periprosthetic fracture (R.R. = 2.14, 95 % C.I. [0.85, 5.38], p = 0.11) between the two approaches, as illustrated in Fig. 6.

#### 3.4.2 Secondary perioperative outcomes

**Fig. 7:**
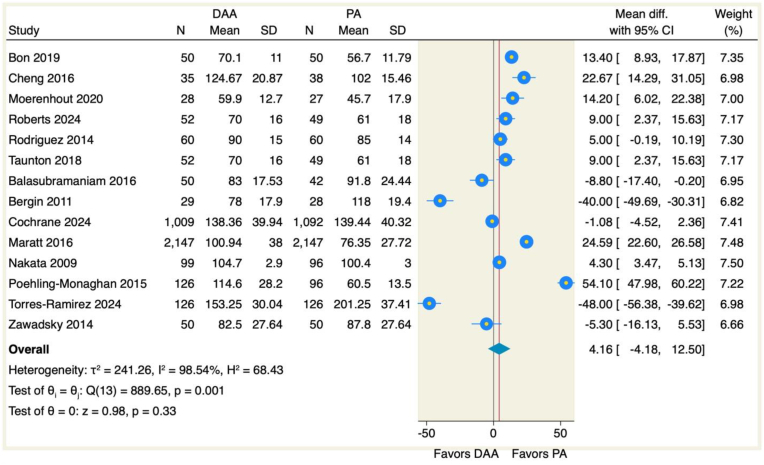
Forest plot of operative time.

The pooling of 14 studies reporting the operative time showed no substantial differences between DAA and PA (M.D. = 4.16 min, 95 % C.I. [−4.18, 12.5], p = 0.33) with associated significant heterogeneity (I^2^ = 98.54 %, p = 0.001) (Fig. 7).

**Fig. 8:**
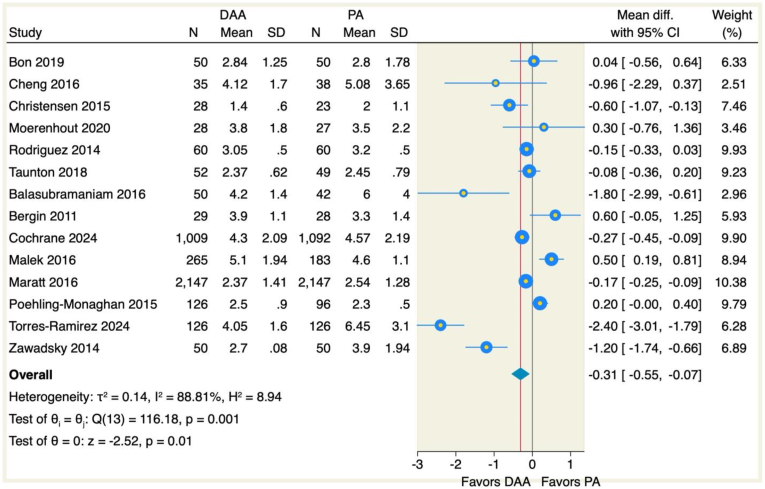
Forest plot of the duration of hospital stay.

Fourteen studies assessed the duration of hospital stay, incorporating 4055 patients in the DAA group and 4011 patients in the PA group. The pooled analysis indicated a significantly shorter hospital stay among patients managed by DAA compared to those who received the PA (M.D. = −0.31 day, 95 % C.I. [−0.55, −0.07], p = 0.01) (Fig. 8). Significant heterogeneity was noticed when pooling the results (I^2^ = 88.81 %, p = 0.001) (Fig. 8).

Although DAA showed a shorter incision length (M.D. = −33.75 mm, 95 % C.I. [−42.97, −24.54], p = 0.001), it demonstrated a higher blood volume loss (M.D. = 99.82 ml, 95 % C.I. [24.07, 175.58], p = 0.01) compared to PA, as illustrated in Supplementary Figs. 5 and 6, respectively. Notably, there was a non-statistically significant difference between the two approaches regarding the need for blood transfusion (R.R. = 0.88, 95 % C.I. [0.74, 1.05], p = 0.17) (Supplementary Fig. 7).

Furthermore, the pooled analysis did not detect a significant variation between the two techniques regarding the discharge to home (R.R. = 1.1, 95 % C.I. [0.98, 1.24], p = 0.1) or the discharge to rehabilitation (R.R. = 0.63, 95 % C.I. [0.38, 1.06], p = 0.08), as outlined in Supplementary Fig. 8.

#### 3.4.3 Secondary functional outcomes

Several functional scores, such as OHS, WOMAC, SF-12, and HHS, were employed to assess the functional outcomes across the included studies. Similar functional recovery was found between DAA and PA in terms of OHS (M.D. = −0.84, 95 % C.I. [−7.08, 5.4], p = 0.79), WOMAC (M.D. = 0.06, 95 % C.I. [−4.18, 4.29], p = 0.98), SF-12 physical score (M.D. = 0.88, 95 % C.1. [−0.62, 2.39], p = 0.25), SF-12 mental score (M.D. = 0.37, 95 % CI [−1, 1.74], p = 0.60), and HHS (M.D. = 0.58, 95 % C.I. [−3.45, 4.61], p = 0.78), as illustrated in Supplementary Figs. 9–13.

A subgroup analysis according to the follow-up duration showed no statically significant variations between the two approaches in HHS scores at 2 weeks (p = 0.20), 3 weeks (p = 0.56), 6 weeks (p = 0.58), 8 weeks (p = 0.09), 12 weeks (p = 0.19), one year (p = 0.95), and 5 years (p = 0.15), as outlined in Supplementary Fig. 14.

In the same context, the VAS pain score with DAA was non-significantly reduced compared to PA (M.D. = −0.77, 95 % C.I. [−2.26, 0.72], p = 0.31), as illustrated in Supplementary Fig. 15.

#### 3.4.4 Complications

No significant difference was noticed between the two approaches regarding surgical site hematoma (R.R. = 1.32, 95 % C.I. [0.86, 2.02], p = 0.20), and infection (R.R. = 1.58, 95 % C.I. [0.93, 2.68], p = 0.09), as displayed in Supplementary Figs. 16 and 17, respectively.

## 4 Discussion

This is the most updated and extensive systematic review and meta-analysis comparing DAA and PA for THA. The pooled analysis showed similar rates of all-cause surgery revision, or revision due to specific complications, such as infection, dislocation, and periprosthetic fracture. In the same context, no substantial differences between the two techniques were noticed in terms of dislocation, intraoperative fracture, and periprosthetic fracture. Notably, DAA was associated with a significant reduction in hospital stay compared to PA. Additionally, the DAA demonstrated shorter incision length but was linked to higher blood loss than PA. However, there was no substantial difference between the two approaches regarding operative time. Similarly, no significant variations between DAA and PA were found in the functional outcomes measured using OHS, WOMAC, SF-12, and HHS scores.

Based on the National Institute for Health and Care Excellence (NICE) recommendations in 2020, the ideal surgical approach for THA remains undetermined. The selection of the surgical approach usually depends on the surgeon's expertise and patient-centred factors rather than evidence-based guidance.^9^ In this context, our study provides valuable insights by comparing one of the most utilized approaches for THA, DAA, and PA. This meta-analysis shows a substantial relevance to the recent evidence from a larger network analysis of 63 RCTs comprising 4859 patients, evaluating eight different surgical modalities for THA, involving DAA and PA.^47^ This study found a significant improvement in hip scores for most approaches, encompassing DAA and PA, compared to the direct lateral approach (DLA). Notably, PA showed a shorter operation time than other approaches, including DAA (MD = −13.94 min, 95 % CI [−18.79, −9.08]). No significant differences were found regarding complication rates among the approaches. Although this network meta-analysis provides a robust comparative effectiveness between multiple approaches, it does not offer a detailed direct comparison of DAA versus PA. Including 27 studies incorporating 44,477 patients, we are confident that our research could fill a critical knowledge gap by directly comparing the two approaches regarding perioperative, functional, and safety outcomes.

Our pooled analysis showed comparable estimates of all-cause surgery revision, dislocation, and fractures between DAA and PA, highlighting that DAA is as effective as PA in preventing major complications following THA. In addition to these comparable complication rates, DAA surpassed PA in terms of perioperative outcomes, as it showed a shorter hospital stay and shorter incision length than PA. DAA is a true intermuscular technique performed via the Heuter approach, which enters the hip through the muscle gap among the rectus femoris, sartorius, and lumbar fascia muscles.^48^ This approach provides adequate maintenance of hip joint stability, as it is performed in the supine position with intraoperative fluoroscopy guidance that ensures adequate prosthesis placement.^12^ DAA was also linked to lower soft tissue damage, as evidenced by Bergin et al., who found a significantly higher muscle damage indicated by high serum CK levels in the PA group compared to DAA.^11^ These observations emphasize the DAA as an effective alternative to PA, with similar safety and functional outcomes and better perioperative results.

However, DAA showed a significantly higher blood loss compared to PA. It could be attributed to several factors related to DAA. First, the DAA was performed in a supine position, which lacks the gravitational advantage in blood drainage provided by the lateral position in PA. Second, the DAA is technically more challenging than PA, particularly in exposing and preparing the femur, which may heighten the bleeding risk. Third, the steep learning curve for DAA noticed in some studies could result in longer femoral preparation and more soft tissue retraction, thus increasing the risk of bleeding from bone and surrounding tissue.^35^

Consequently, the minimally invasive nature of DAA, which avoids muscle splitting and produces minimal soft tissue damage, may also enhance the early functional recovery. A meta-analysis encompassing 9 RCTs with 754 THAs found better early functional outcomes with DAA, indicated by the higher HHS scores at 2 and 4 weeks postoperatively, compared to the PA.^13^ However, our meta-analysis did not show any greater benefit for DAA over PA in improving the functional outcomes, either in the early or long-term follow-up period. These notable variations between studies could be attributed to the differences in the included studies, patient characteristics, and methodological approaches. Notably, the early functional benefit noticed in Wang et al. study also diminished after 12 weeks. These consistent long-term results across both studies indicate that while DAA may provide early functional benefits during the early recovery phase, it does not show any superior benefit in the long-term function when compared to PA.

Notably, our pooled analysis showed comparable intraoperative fracture rates between the two approaches. In contrast, A previous meta-analysis by Jia et al.^12^ suggested a higher risk of intraoperative fractures with DAA than PAA. It is worth noting that the higher incidence of intraoperative fractures could be attributed to the restriction of intraoperative positioning, which necessitates greater surgical precision and may challenge less experienced surgeons.^12^ Furthermore, nerve injury is another recognized complication of DAA, particularly the neuropraxia of the cutaneous nerve of the thigh.[49,51] However, Goulding et al.^49^ revealed that this nerve injury does not significantly affect postoperative functional recovery, varies considerably among surgeons, and may depend on their level of expertise.

The learning curve of DAA has been identified as a significant factor impacting postoperative complication rates. Emerging evidence showed an elevated complication rate for DAA during the early learning phase.^50^^,^^51^ However, D'Arrigo et al.^52^ further noted that the learning curve of DAA could increase the surgical time, but it did not impact THA's efficacy or safety profile. These contradictory results highlight the ongoing debate concerning the influence of the learning curve of DAA on surgical outcomes. Further high-quality RCTs are recommended to address this conflict. Additionally, patient-centred factors could also affect the risk of complications. Particularly, Male patients and individuals with elevated BMI were associated with substantial challenges during DAA, which could heighten the risk of intraoperative and postoperative complications.^53^

### 4.1 Strengths and limitations

This systematic review and meta-analysis represent the most up-to-date evidence evaluating DAA and PA for THA, including 27 studies comprising 44,477 patients. Moreover, including RCTs and observational studies across various geographical areas enhances the generalizability and applicability of our analysis to the whole population. However, like any available research, our study encounters several limitations. First, including observational studies could introduce selection bias and weaken our analysis power. Second, there was a significant heterogeneity in several outcomes that could limit the generalizability and mask the true effect estimates. This notable heterogeneity could be explained by the substantial differences in population characteristics, surgeons’ experience, and clinical settings. Third, the limited studies from non-Western countries could affect the global generalizability of the findings.

### 4.2 Implications in clinical practice and future suggestions

This meta-analysis emphasizes the efficacy of DAA as an effective alternative to PA for patients undergoing THA, owing to its shorter hospital stay and minimally invasive nature. Furthermore, the application of DAA may be feasible in facilities equipped for supine positioning and intraoperative fluoroscopy. Nevertheless, the comparable functional outcomes and complications between the two approaches limit the recommendation of DAA over PA, highlighting that surgeon expertise and individual-specific factors should remain the main determinants of approach selection. Future researchers should conduct high-quality multicenter trials with long-term follow-up to provide robust evidence regarding the long-term functionality of DAA. Furthermore, additional studies from the underrepresented regions, especially Asia and Africa, are needed to optimize the generalizability.

## 5 Conclusion

This meta-analysis highlights the similar efficacy of DAA compared to PA in preventing major complications following THA, including all-cause surgery revision, dislocation, and fracture. Notably, DAA was linked to shorter hospital stays and incision lengths compared to PA. Additionally, both approaches revealed comparable short and long-term functional recovery. More long-term studies from underrepresented regions are required to support our findings and ensure the selection process of the optimal surgical approach.

## CRediT authorship contribution statement

**Maher Ghandour:** Conceptualization, Supervision, Project administration, and was responsible for manuscript review. **Ouriel Salomon:** Methodology, Formal analysis, screening, data extraction, quality assessment, Data curation, Writing – original draft, Visualization. **Massinissa Hammouchi:** Investigation, Resources, Writing – review & editing. **Aboubacar Lawan Abdou:** Investigation, Formal analysis, and manuscript review. **Kevin Okoma:** Investigation, Formal analysis, and manuscript review. **Meriem Souissi:** Investigation, Formal analysis, and manuscript review. **Dounia Brahimi:** Data curation, quality assessment, Writing – review & editing. **Lisa Gosnave:** Data curation, quality assessment, Writing – review & editing. **Ümit Mert:** Supervision, Validation, and critical revision of the manuscript. **Mauz Asghar:** Methodology, screening, data extraction, quality assessment, Validation, Writing – review & editing. **Julien Mayer:** Data curation, quality assessment, Writing – review & editing. **Emmanuel Caremier:** Resources, Supervision, and contributed to quality assessment and manuscript revision. **Aboubekr Berrichi:** Investigation, Resources, screening, and manuscript review. **M'barek Irrazi:** Investigation, Resources, screening, and manuscript review.

## Guardian/patient's consent

N/A.

## Patient involvement

No.

## Guardian/patient's consent

N/A.

## Contributorship

N/A.

## Clinical trial number

N/A.

## Availability of data and materials

All data generated or analysed during this study are included in this published article [and its supplementary information files].

## Consent for publication

N/A.

## Data sharing statement

N/A.

## Ethical approval information

N/A.

## Ethical statement

N/A.

## Funding statement

The study is not funded by any grant.

## Conflict of interest

The authors declare that they have no known competing financial interests or personal relationships that could have appeared to influence the work reported in this paper.
