# Supplementary material for: Direct anterior approach versus posterior approach in total hip arthroplasty: A systematic review and meta-analysis
Source: J Orthop. 2025 Jun 14;65:233–50. doi: 10.1016/j.jor.2025.06.007 (PMC12210301; doi:10.1016/j.jor.2025.06.007)
Supplement: Multimedia component 1 [file mmc1.docx]

**Supplementary Table 1:** Search strategy for each database.

| Database | Search term | Filter | Results |
| --- | --- | --- | --- |
| PubMed | (("Direct anterior Approach" OR "DAA" OR "Direct Anterior Minimally Invasive Surgery" OR "DA MIS" OR "Mini-Anterior Approach" OR "Smith-Petersen Approach" OR "Anterior Supine Intermuscular Approach" OR "Anterior-Based Muscle-Sparing Approach" OR "Anterior Intermuscular Approach") AND ("posterior approach" OR "PA" OR "Dorsal approach" OR "Posterior access" OR "Posterior incision" OR "Retrograde approach") AND ("Total Hip Replacements" OR "THR" OR "Hip Joint Replacement" OR "Hip Prosthesis Surgery" OR "Hip Arthroplasty" OR "Total Hip Prosthesis" OR "Hip Reconstruction Surgery" OR "Artificial Hip Implant")) | All  Fields | 237 |
| WOS | (("Direct anterior Approach" OR "DAA" OR "Direct Anterior Minimally Invasive Surgery" OR "DA MIS" OR "Mini-Anterior Approach" OR "Smith-Petersen Approach" OR "Anterior Supine Intermuscular Approach" OR "Anterior-Based Muscle-Sparing Approach" OR "Anterior Intermuscular Approach") AND ("posterior approach" OR "PA" OR "Dorsal approach" OR "Posterior access" OR "Posterior incision" OR "Retrograde approach") AND ("Total Hip Replacements" OR "THR" OR "Hip Joint Replacement" OR "Hip Prosthesis Surgery" OR "Hip Arthroplasty" OR "Total Hip Prosthesis" OR "Hip Reconstruction Surgery" OR "Artificial Hip Implant")) | All fields | 381 |
| Cochrane  Central | (("Direct anterior Approach" OR "DAA" OR "Direct Anterior Minimally Invasive Surgery" OR "DA MIS" OR "Mini-Anterior Approach" OR "Smith-Petersen Approach" OR "Anterior Supine Intermuscular Approach" OR "Anterior-Based Muscle-Sparing Approach" OR "Anterior Intermuscular Approach") AND ("posterior approach" OR "PA" OR "Dorsal approach" OR "Posterior access" OR "Posterior incision" OR "Retrograde approach") AND ("Total Hip Replacements" OR "THR" OR "Hip Joint Replacement" OR "Hip Prosthesis Surgery" OR "Hip Arthroplasty" OR "Total Hip Prosthesis" OR "Hip Reconstruction Surgery" OR "Artificial Hip Implant")) | All Fields | 33 |
| Scopus | (("Direct anterior Approach" OR "DAA" OR "Direct Anterior Minimally Invasive Surgery" OR "DA MIS" OR "Mini-Anterior Approach" OR "Smith-Petersen Approach" OR "Anterior Supine Intermuscular Approach" OR "Anterior-Based Muscle-Sparing Approach" OR "Anterior Intermuscular Approach") AND ("posterior approach" OR "PA" OR "Dorsal approach" OR "Posterior access" OR "Posterior incision" OR "Retrograde approach") AND ("Total Hip Replacements" OR "THR" OR "Hip Joint Replacement" OR "Hip Prosthesis Surgery" OR "Hip Arthroplasty" OR "Total Hip Prosthesis" OR "Hip Reconstruction Surgery" OR "Artificial Hip Implant")) | All Fields | 231 |


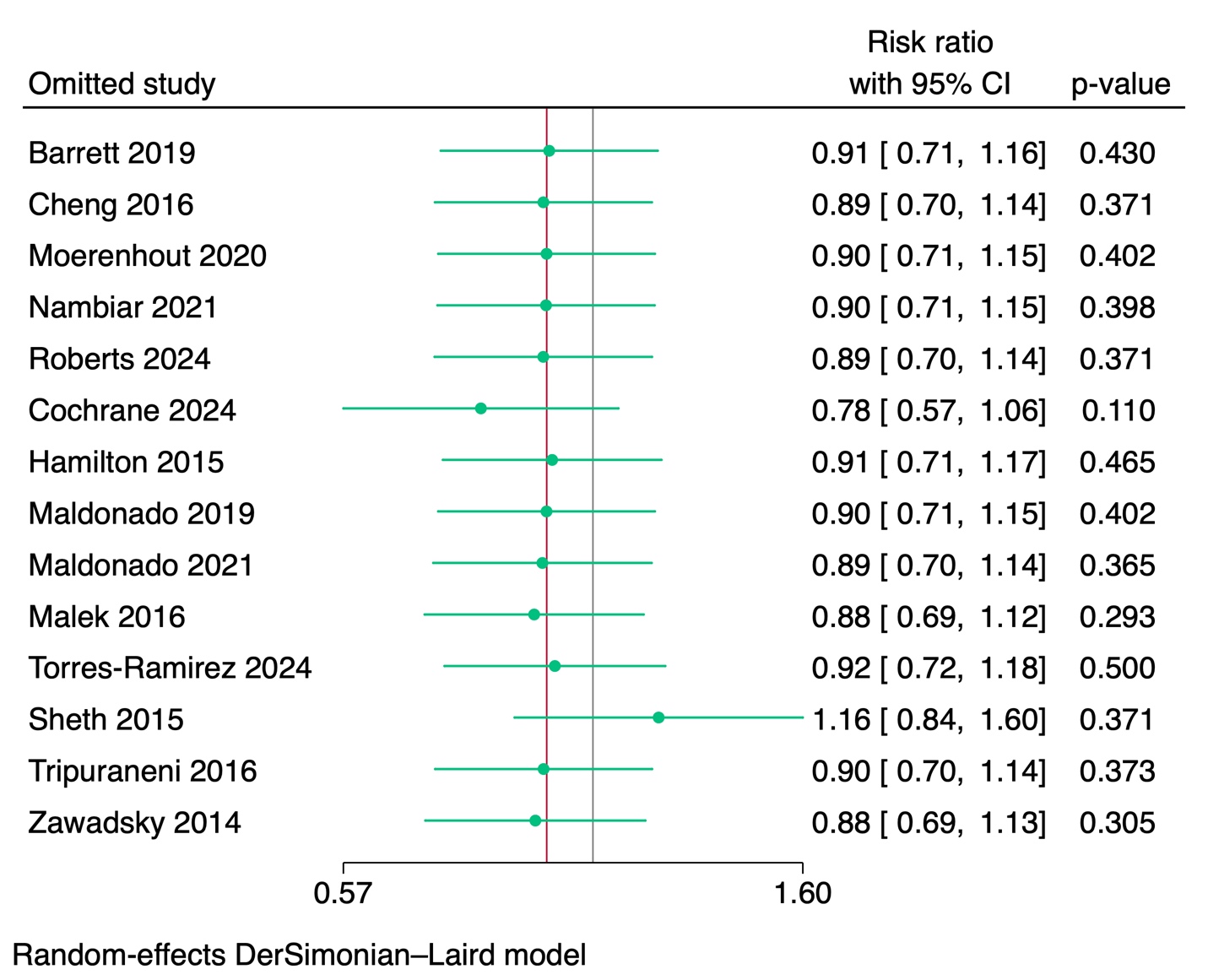


**Supplementary Figure 1:** Leave-one-out test of all-cause surgery revision.


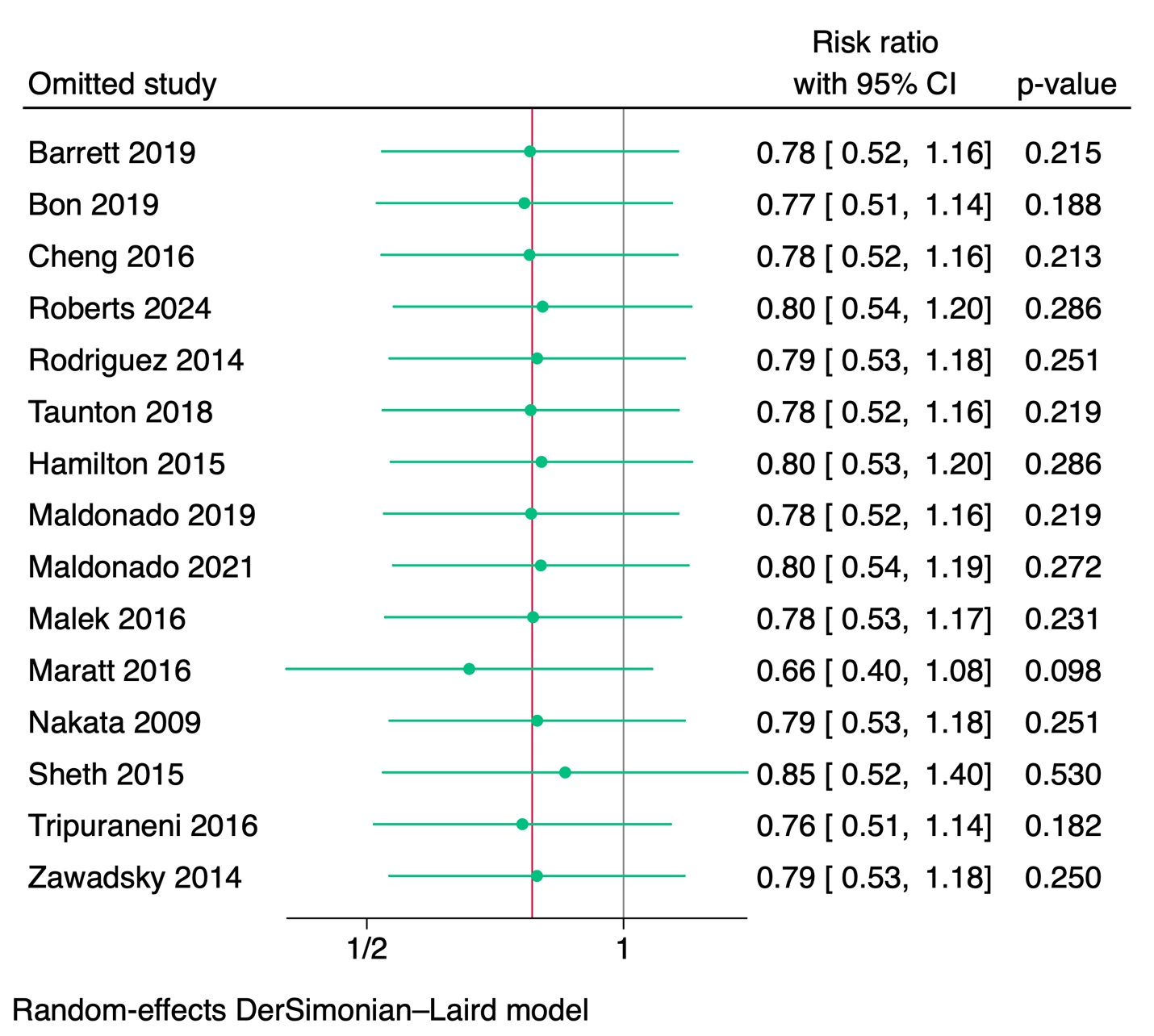


**Supplementary Figure 2:** Leave-one-out test of dislocation.


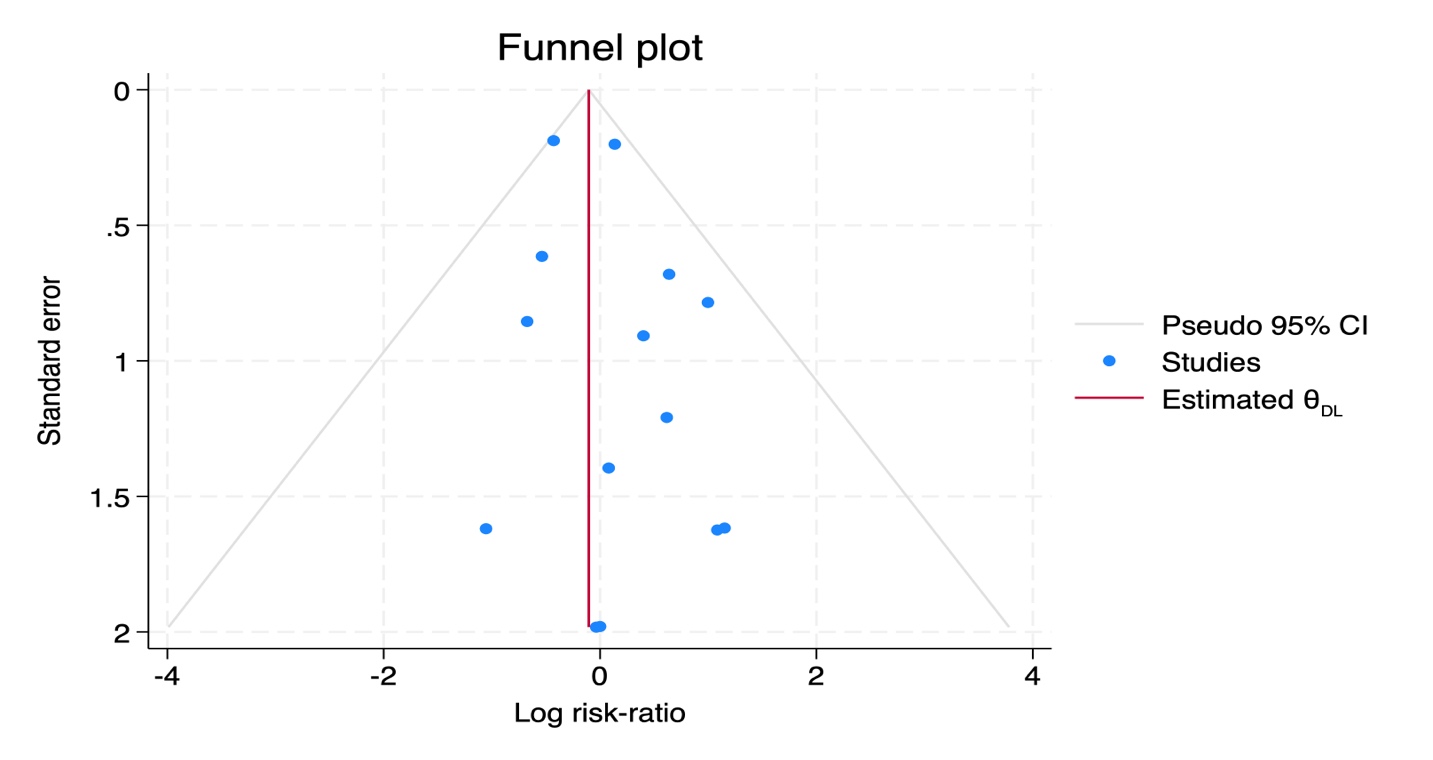
**Supplementary Figure 3:** Funnel plot of all-cause surgery revision.


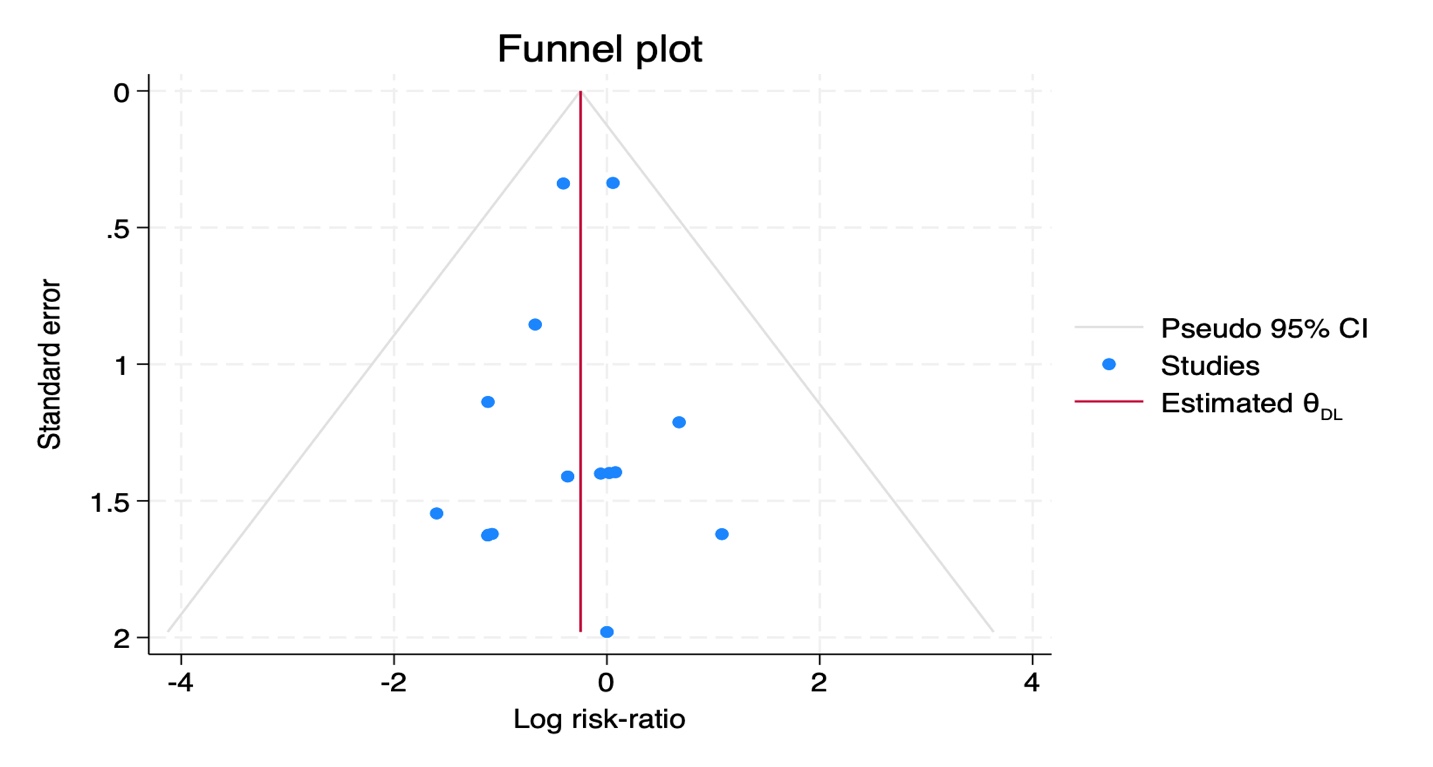
**Supplementary Figure 4:** Funnel plot of dislocation.


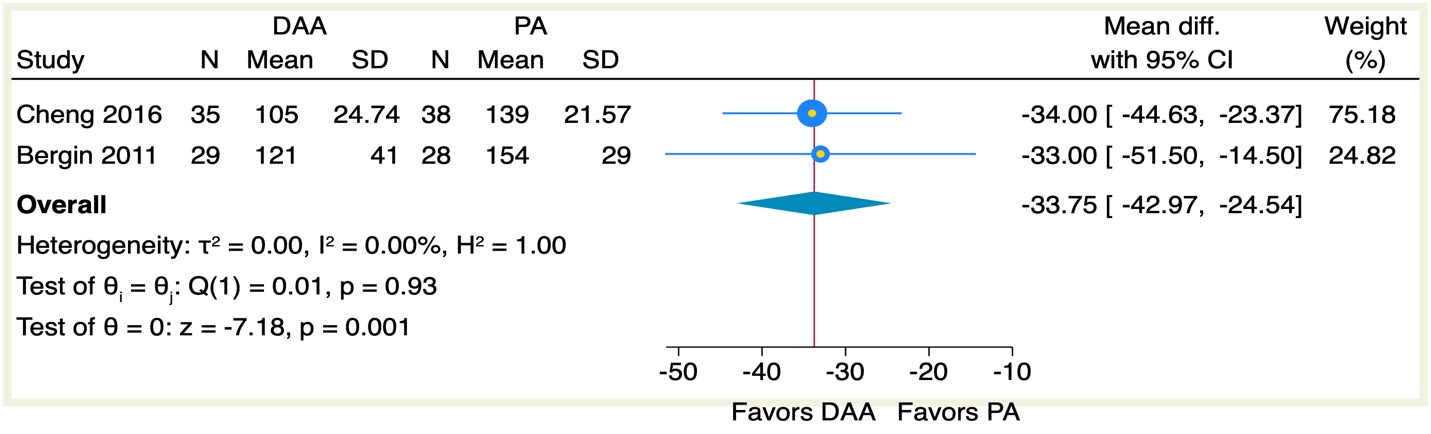


**Supplementary Figure 5:** Forest plot of incision length.


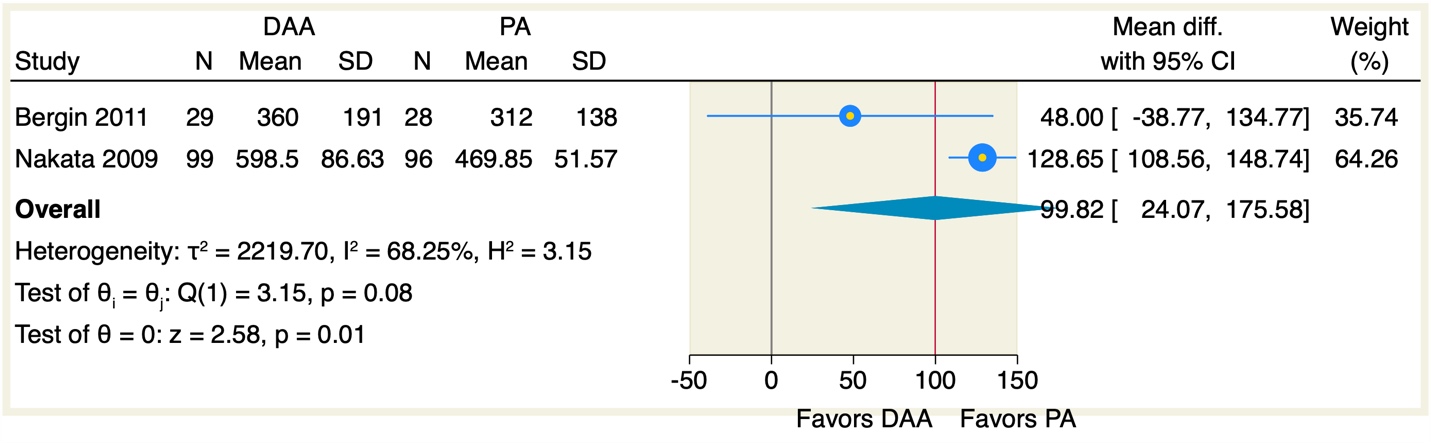


**Supplementary Figure 6:** Forest plot of blood volume loss.


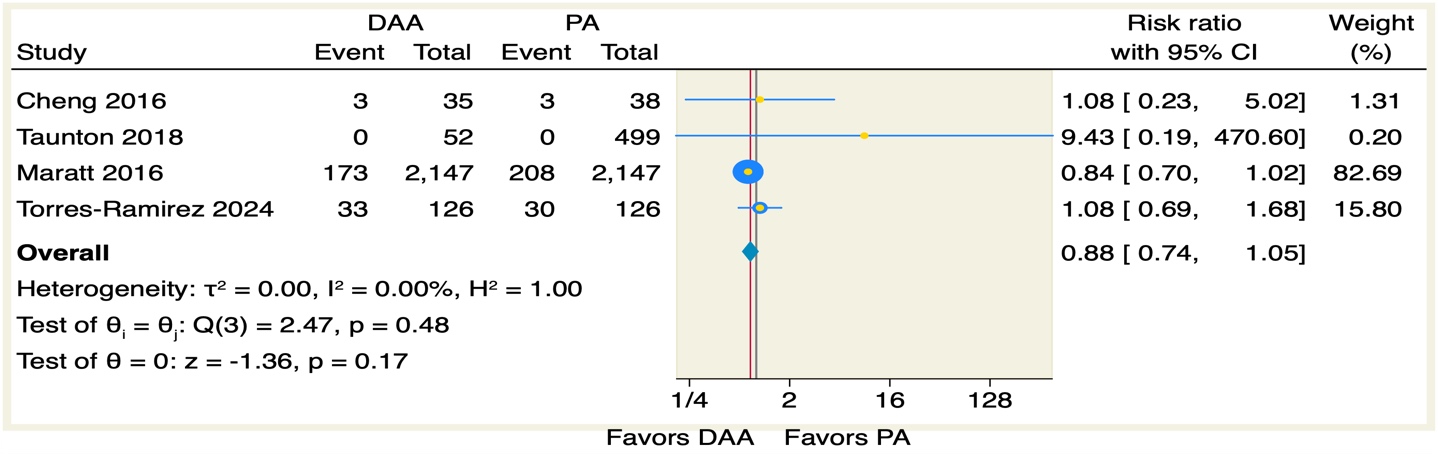


**Supplementary Figure 7:** Forest plot of the need for blood transfusion.


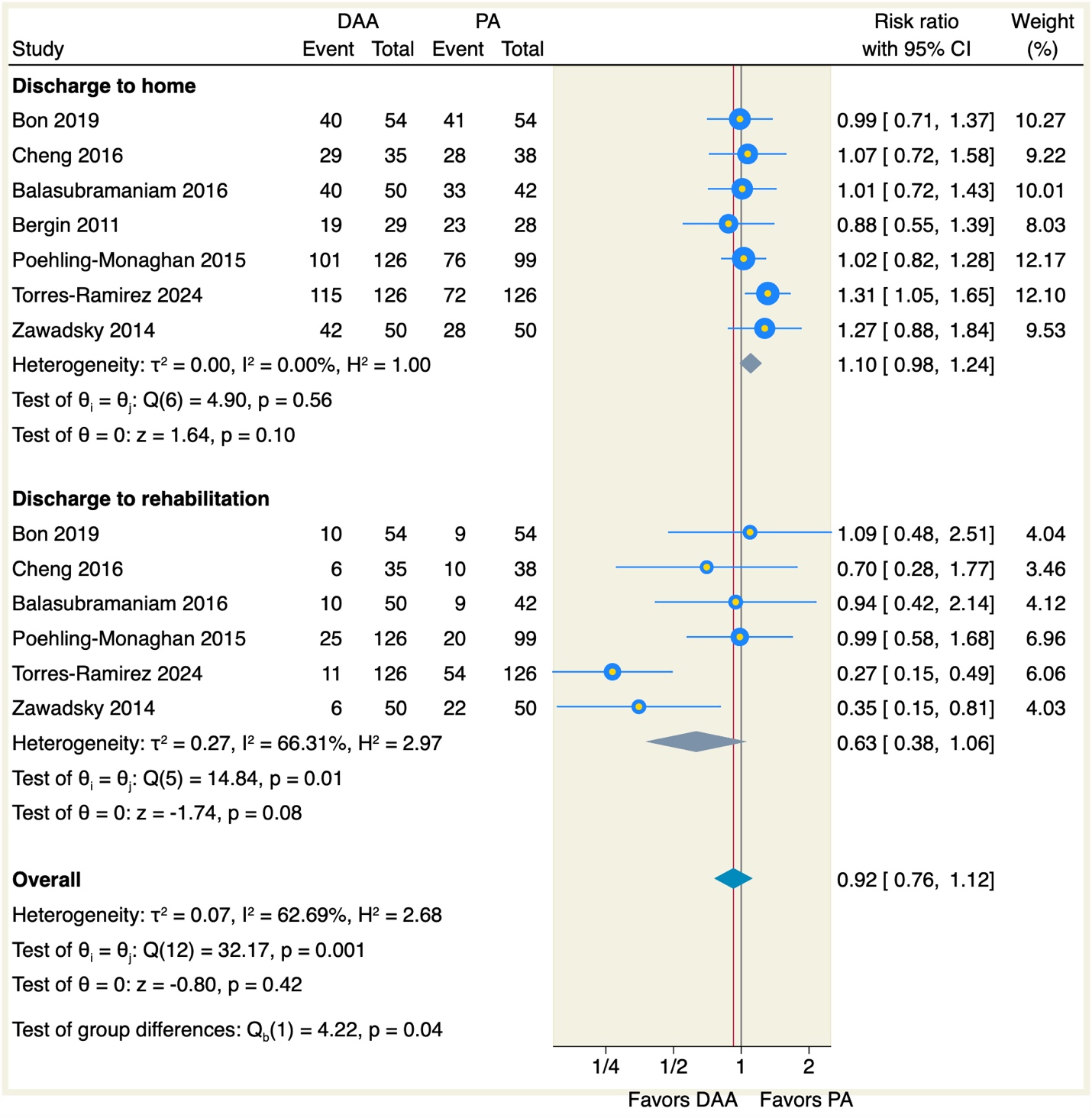


**Supplementary Figure 8:** Forest plot of discharge.


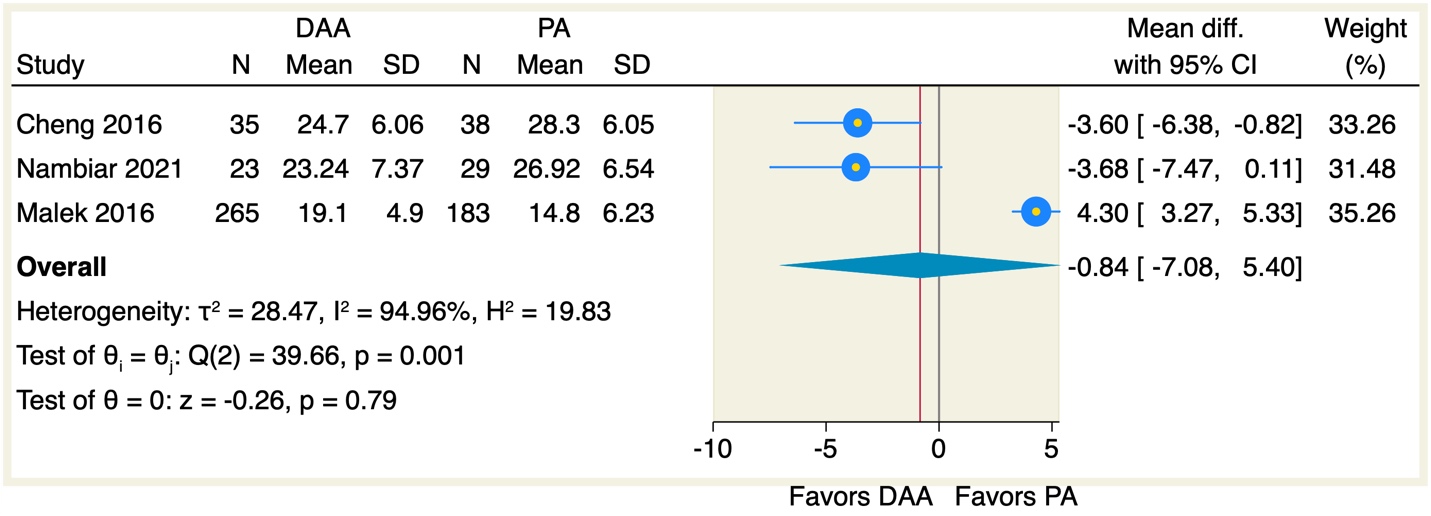
**Supplementary Figure 9:** Forest plot of Oxford Hip Score.


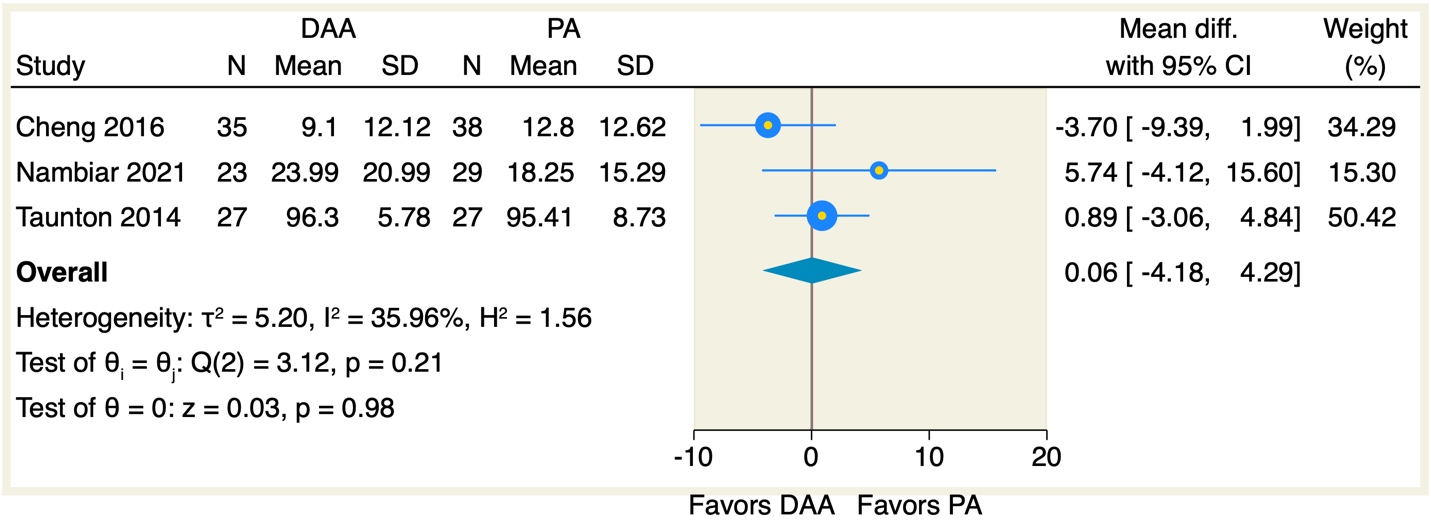


**Supplementary Figure 10:** Forest plot of Western Ontario and McMaster Universities Osteoarthritis.


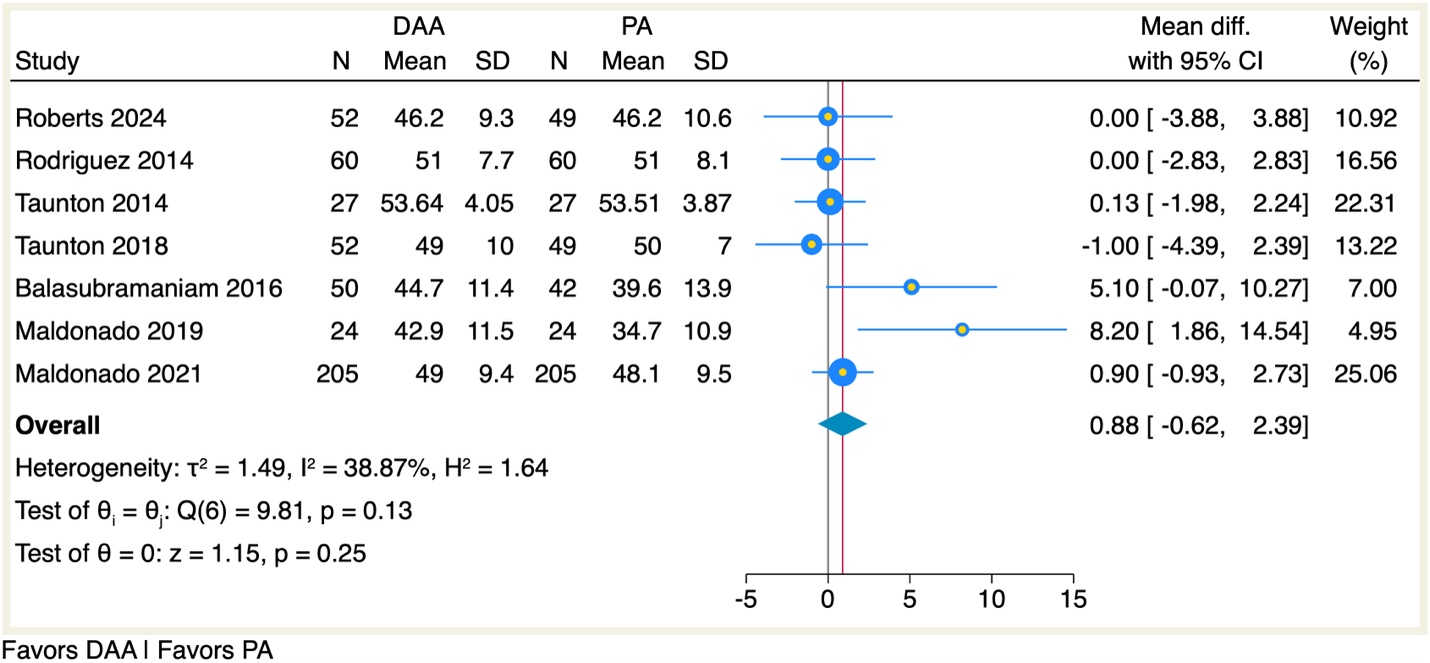


**Supplementary Figure 11:** Forest plot of Health Survey Short Forms (SF-12) physical score.


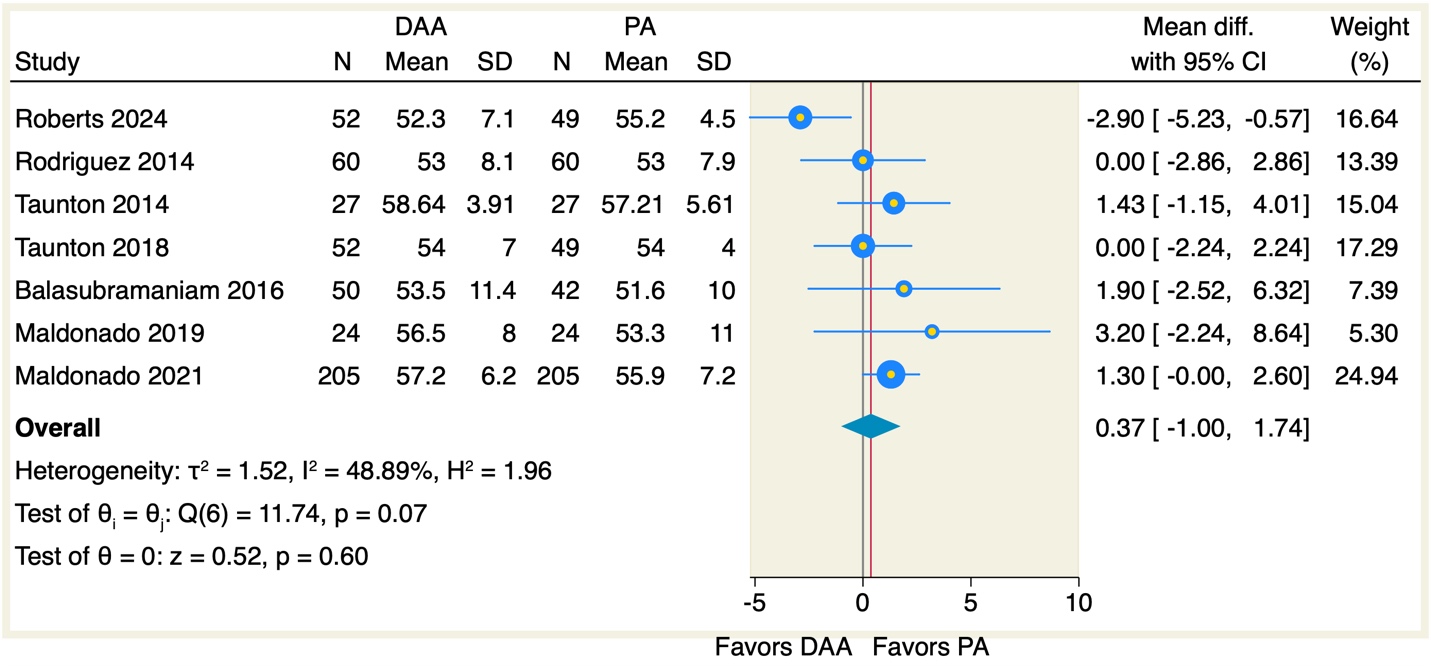


**Supplementary Figure 12:** Forest plot of Health Survey Short Forms (SF-12) mental score.


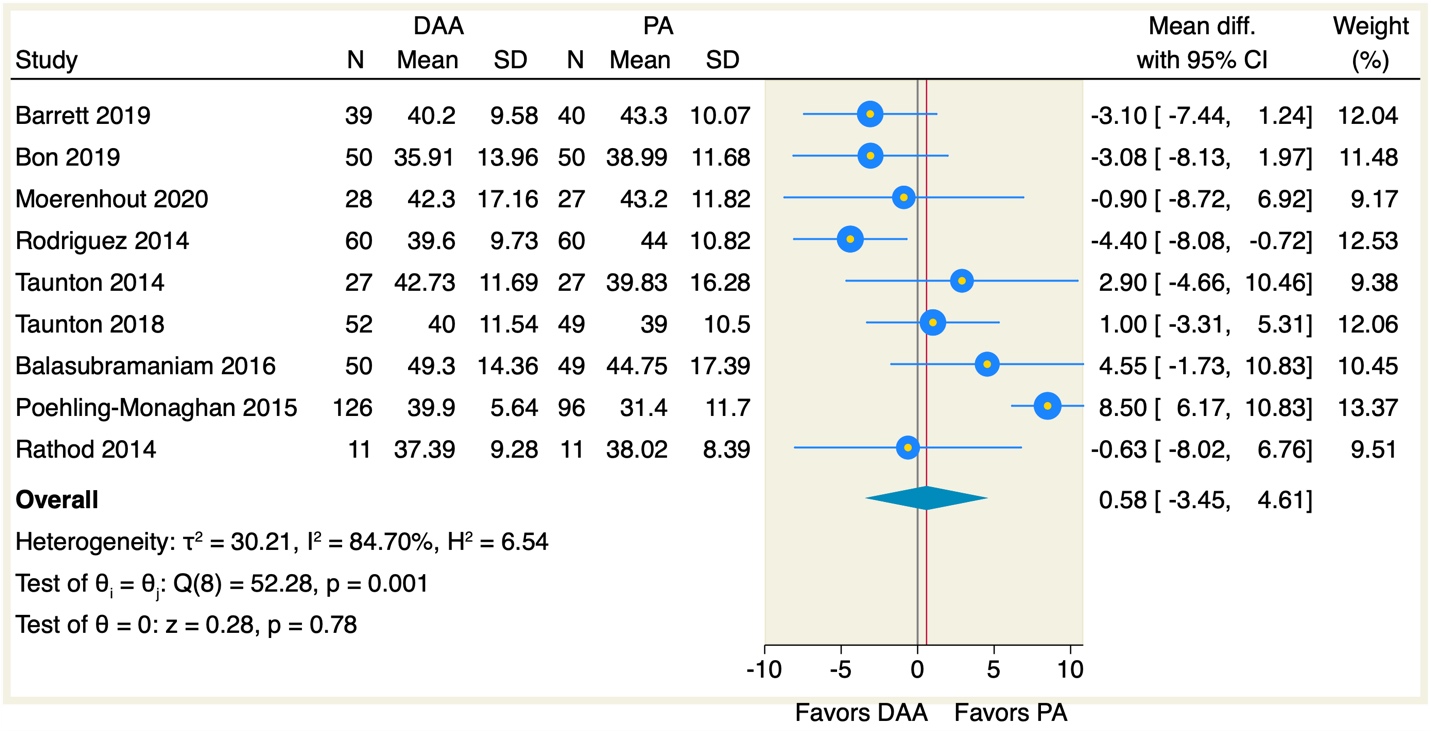


**Supplementary Figure 13:** Forest plot of Harris Hip Score.


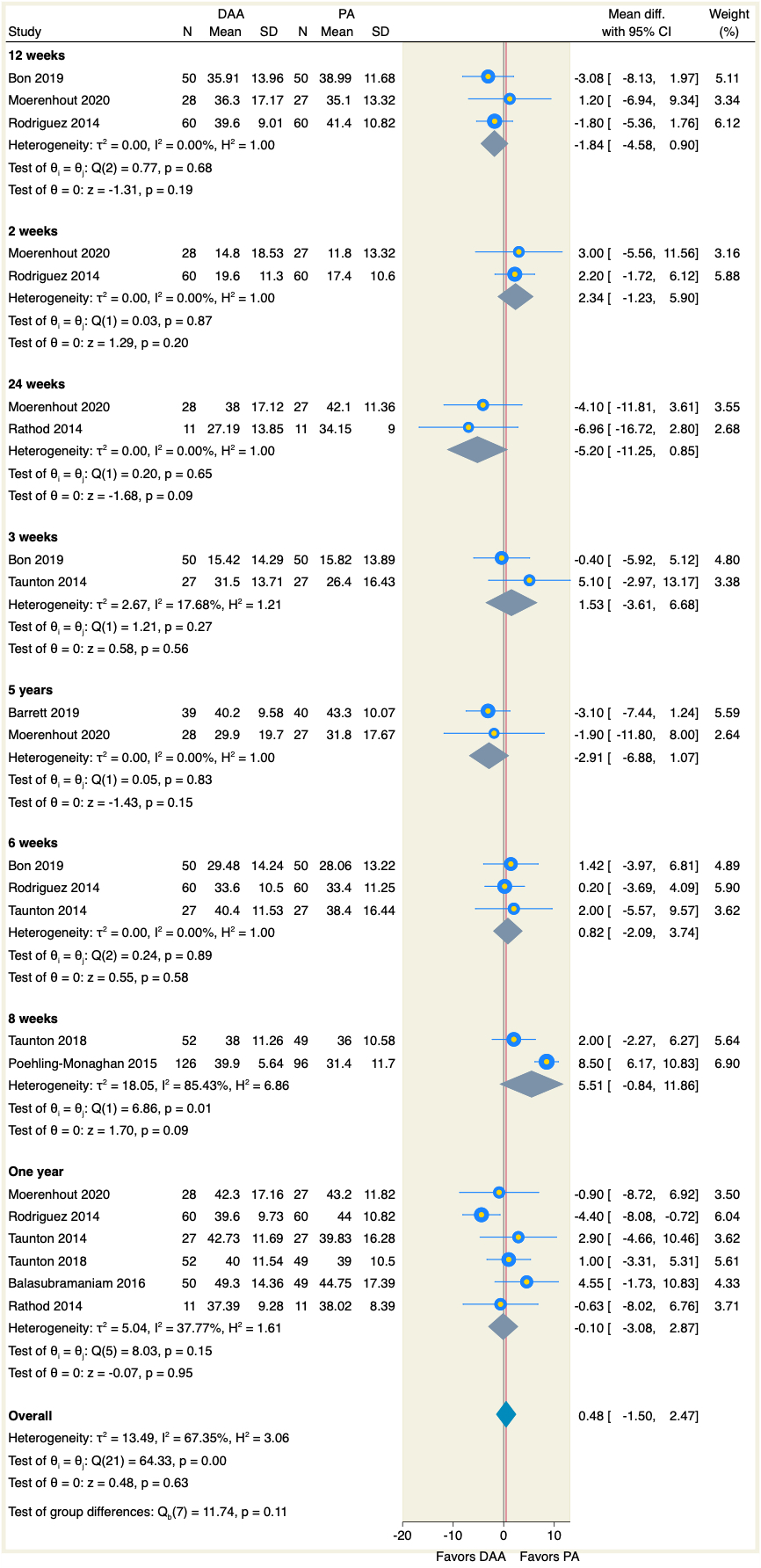


**Supplementary Figure 14:** Forest plot of Harris Hip Score stratified according to the follow-up period.


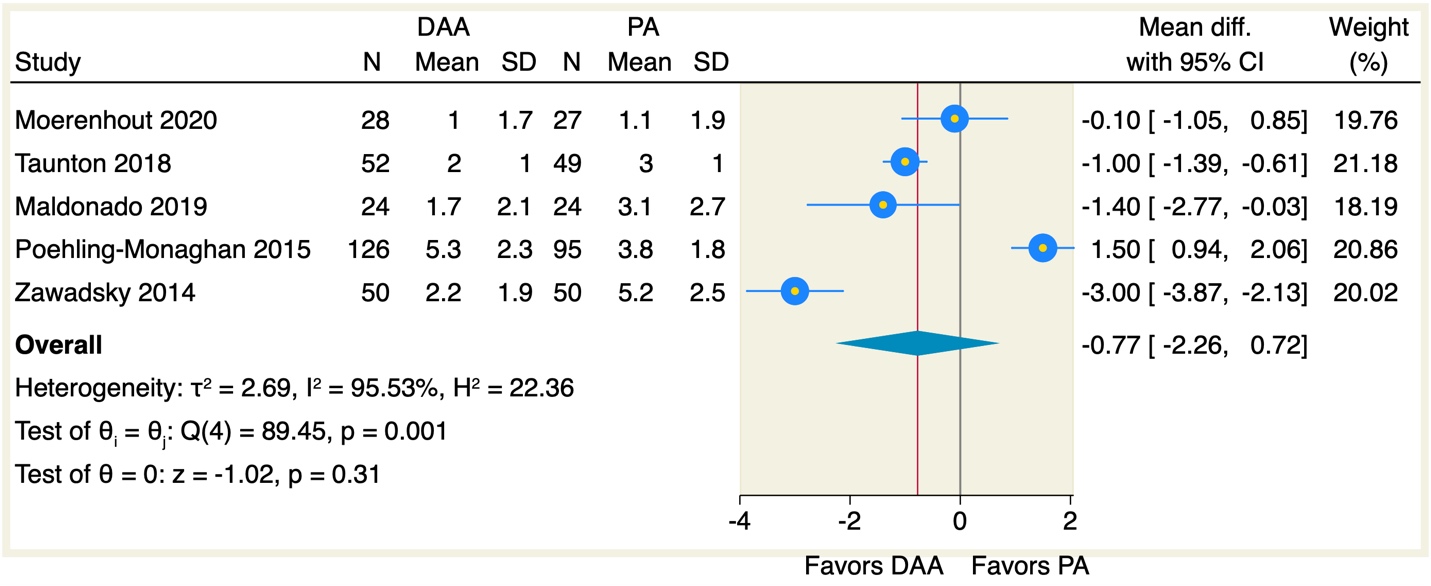


**Supplementary Figure 15:** Forest plot of pain measured by visual analogue scale.


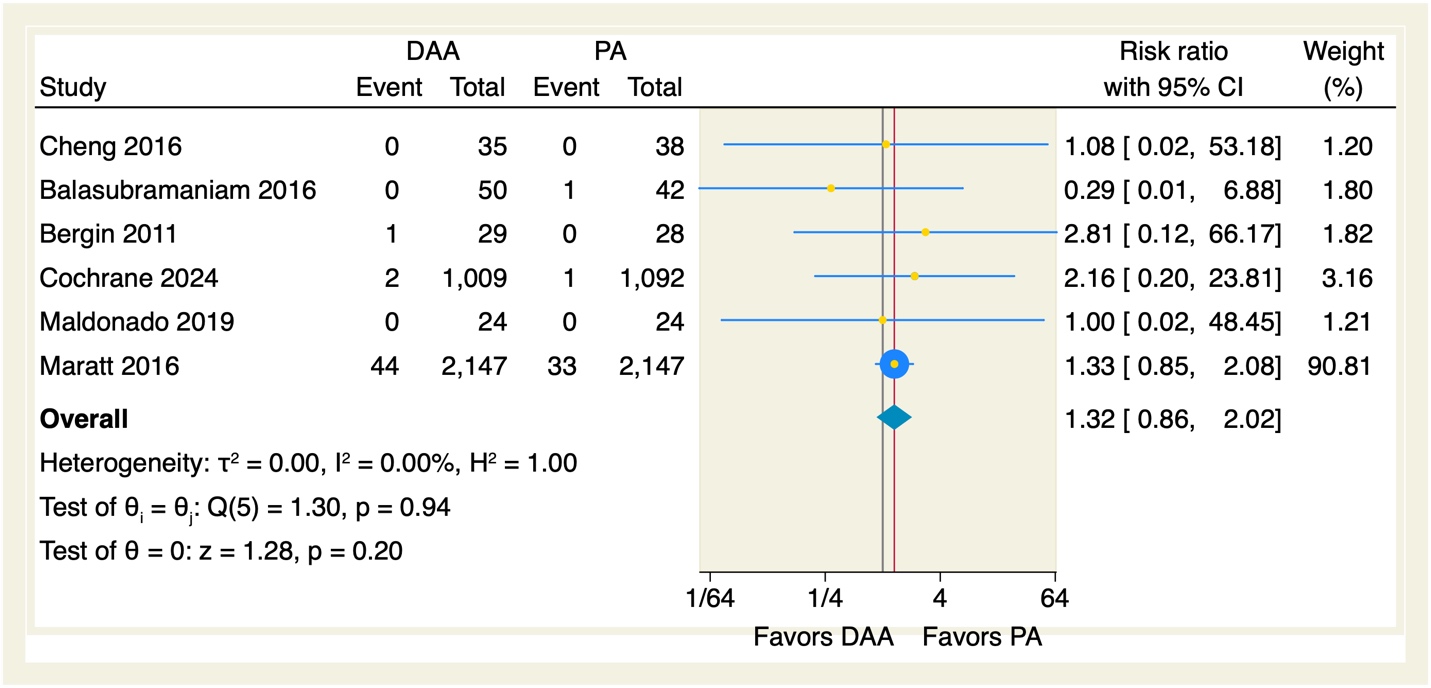


**Supplementary Figure 16:** Forest plot of surgical site hematoma.


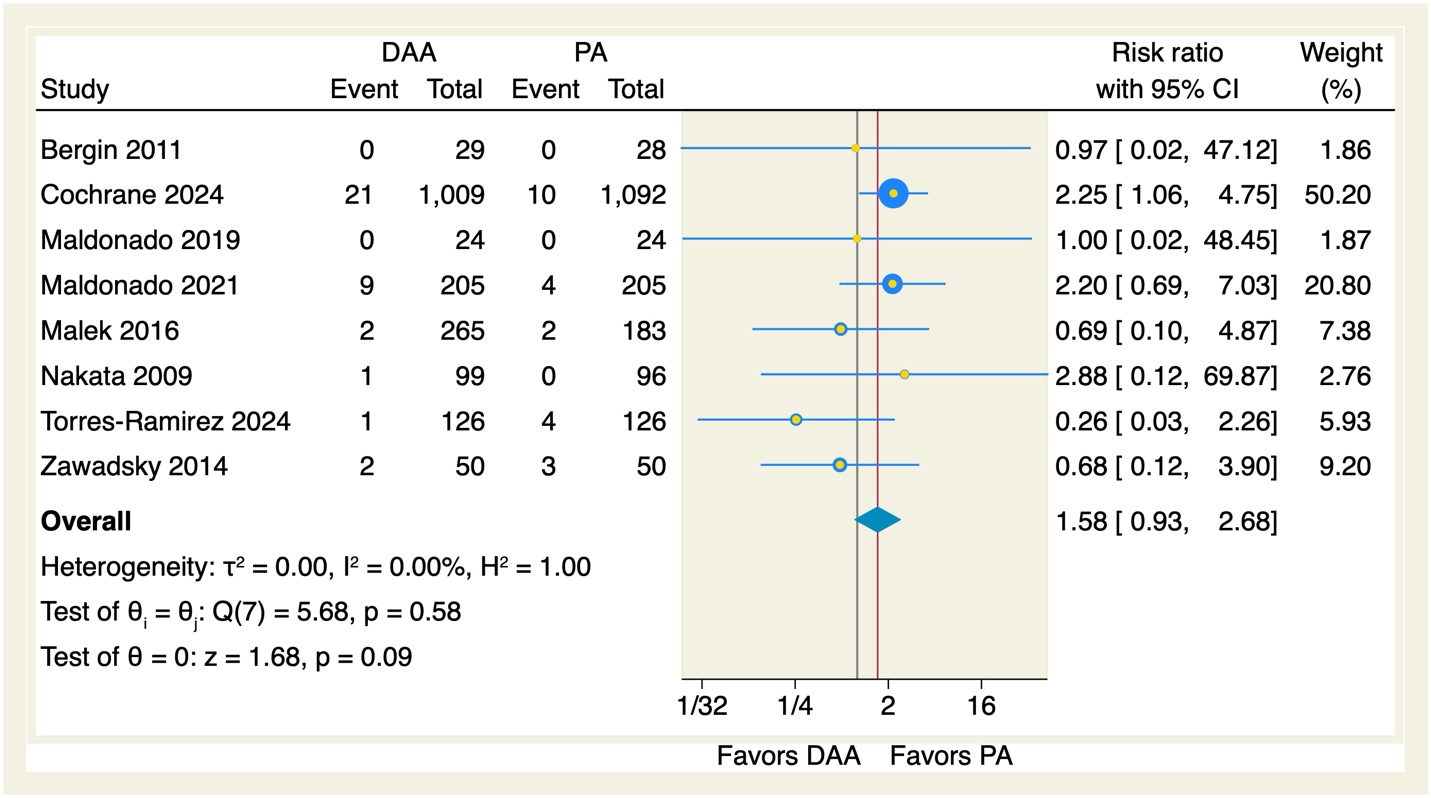


**Supplementary Figure 17:** Forest plot of infection.
